# Transketolase regulates sensitivity to APR-246 in p53-null cells independently of oxidative stress modulation

**DOI:** 10.1038/s41598-021-83979-3

**Published:** 2021-02-24

**Authors:** Julia V. Milne, Bonnie Z. Zhang, Kenji M. Fujihara, Swati Dawar, Wayne A. Phillips, Nicholas J. Clemons

**Affiliations:** 1 Peter MacCallum Cancer Centre, 305 Grattan St, Melbourne, VIC 3000 Australia; 2 Sir Peter MacCallum Department of Oncology, The University of Melbourne, Parkville, VIC 3010 Australia; 3 Department of Surgery (St Vincent’s Hospital), The University of Melbourne, Parkville, VIC 3010 Australia

**Keywords:** Cancer, Cancer therapy, Targeted therapies

## Abstract

The prevalence and dire implications of mutations in the tumour suppressor, p53, highlight its appeal as a chemotherapeutic target. We recently showed that impairing cellular antioxidant systems via inhibition of SLC7A11, a component of the system x_c_^−^ cystine-glutamate antiporter, enhances sensitivity to mutant-p53 targeted therapy, APR-246. We investigated whether this synergy extends to other genes, such as those encoding enzymes of the pentose phosphate pathway (PPP). TKT, one of the major enzymes of the PPP, is allegedly regulated by NRF2, which is in turn impaired by accumulated mutant-p53 protein. Therefore, we investigated the relationship between mutant-p53, TKT and sensitivity to APR-246. We found that mutant-p53 does not alter expression of TKT, nor is TKT modulated directly by NRF2, suggesting a more complex mechanism at play. Furthermore, we found that in p53null cells, knockdown of TKT increased sensitivity to APR-246, whilst TKT overexpression conferred resistance to the drug. However, neither permutation elicited any effect on cells overexpressing mutant-p53 protein, despite mediating oxidative stress levels in a similar fashion to that in p53-null cells. In sum, this study has unveiled TKT expression as a determinant for sensitivity to APR-246 in p53-null cells.

## Introduction

The tumour suppressor, p53, the ‘guardian of the genome’, lies at the crux of a complex transcriptional network of proteins involved in maintaining genome integrity^1,2^. Mutation of p53 frequently results in loss of its direct transcriptional ability and deregulation of its tumour suppression pathways^1,3,4^, and can lead to accumulation of mutant p53 protein with gain-of-function oncogenic activity^5–11^.

Targeting mutant or otherwise deregulated p53 is an attractive avenue for cancer therapeutics due to the frequency of such permutations in cancer. As such, numerous strategies have been developed to reactivate mut-p53, including inducing conformational changes in mut-p53 to restore its wild-type function. This effect has been demonstrated with the use of the first-in-class small molecule drug, APR-246 (also known as PRIMA-1^Met^)^12^, which is currently showing promise in clinical trials^13^.

APR-246 is hydrolysed into its active form, methylene quinuclidinone (MQ), inside cells where it interacts directly with cysteine residues in the p53 protein^4,12,14^. A subsequent change to the mutant p53 protein conformation restores its tumour-suppressing functions, namely induction of apoptosis via transactivation of target genes *PUMA*, *NOXA* and *BAX*^12,15^. APR-246 potentially also abrogates any oncogenic gain-of-function activity acquired by mut-p53.

In addition, APR-246 has been found to interfere with cellular antioxidant pathways involving glutathione and thioredoxin reductase^16–18^ by the formation of adducts between MQ and cysteine^15^ or selenocysteine^17^, respectively, thereby reducing the antioxidant capacities of the cell. Thus, APR-246 encourages an unfavourably high oxidative stress environment that ultimately results in induction of cell death pathways^15–17^. Importantly, accumulation of mut-p53 protein interferes with the cellular redox response through impairment of the key transcription factor nuclear factor (erythroid-derived 2)-like 2 (NRF2)^11,16,19–21^, which regulates expression of many antioxidant-related genes including thioredoxin reductase and key enzymes in the de novo glutathione synthesis pathway. Thus, mutant p53 itself provides cell selectivity for the oxidative stress-mediated effects of APR-246 through promoting a pro-oxidative state within the cell and/or impairing feedback responses that combat oxidative stress^19,20^.

NADPH is a crucial factor in the recycling of cellular antioxidants such as the enzymatic reduction of oxidised glutathione (GSSG) to reduced glutathione (GSH) by glutathione reductase, and also in the recycling of thioredoxin via thioredoxin reductase^17,22,23^. One of the major sources of NADPH in the cell is the pentose phosphate pathway (PPP)^24,25^. Transketolase (TKT), encoded by the *TKT* gene, is a multifunctional enzyme in the non-oxidative arm of the PPP that serves to produce ribose-5-phosphate, a precursor molecule for nucleotide synthesis, as well as channelling its intermediates back into glycolysis in response to oxidative stress and demand for NADPH^25^.

It has been proposed that *TKT* expression, like many other antioxidant-related genes, is regulated by NRF2^25,26^ and *TKT* is overexpressed in many tumour types^27^. It follows that TKT has been found to influence the cellular redox balance, where its knockdown causes an accumulation of intracellular ROS, sensitising tumour cells to radio- and chemotherapies^25^. Therefore, we hypothesised that (i) TKT levels would be regulated by accumulation of mut-p53 through its interaction with NRF2, and (ii) that TKT expression would be an important consideration in determining response to APR-246.

## Results

### TKT is not regulated by mutant-p53 or NRF2 activation

**Figure 1:**
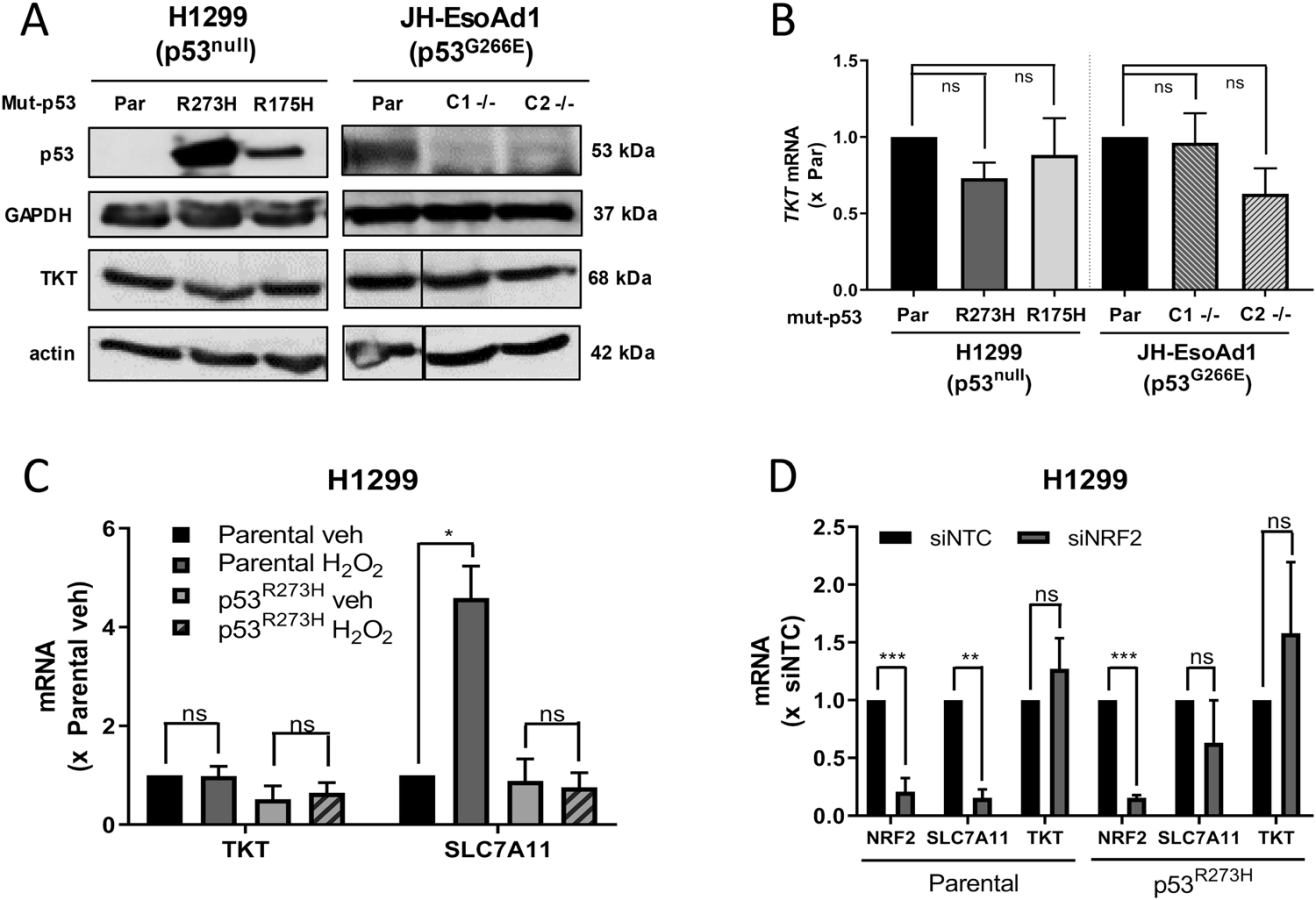
TKT is not regulated by mutant-p53 or NRF2 activation. (**A**) Expression of p53 and TKT protein in H1299 and JH-EsoAd1 cells (Par, parental; C1 −/−, p53−/− clone 1; C2 −/−, p53−/− clone 2). (**B**) *TKT* mRNA (bottom) in H1299 and JH-EsoAd1 cells, with mRNA expression normalised to parental cells (Par, parental; C1 −/−, p53−/− clone 1; C2 −/−, p53−/− clone 2). (**C**) *TKT* and *SLC7A11* mRNA expression in H1299 parental (p53^null^) and p53^R273H^ cells following treatment with hydrogen peroxide (H_2_O_2_, 50 µM) for 3 h, normalised to parental vehicle treatment. (**D**) *NRF2*, *SLC7A11* and *TKT* mRNA expression in H1299 parental (p53^null^) and p53^R273H^ cells following 48 h *NRF2* knockdown with siRNA, normalised to siNTC (siNTC, non-targeting control; siNRF2, *NRF2* siRNA). Data represent mean, error bars = SEM, n ≥ 3 independent experiments for all studies excluding (**A**) where blots are representative of 2 independent experiments. (**B**,**C**) Randomised block one-way ANOVA on raw data with Greenhouse–Geisser correction and Dunnett’s multiple comparison post-test. (**D**) Unpaired Student’s *t*-test on raw data. ns, not significant; *p < 0.05; **p < 0.01; ***p < 0.001.

To investigate the effect of mut-p53 on transketolase (TKT) expression, two isogenic cell systems were used. H1299 cells, a non-small cell lung cancer line, harbour a mutation that prevents the formation of full-length active p53 protein^28^. These cells were previously transduced to ectopically overexpress common missense mutant p53 proteins, p53^R273H^ or p53^R175H^^16^. JH-EsoAd1 cells are an oesophageal cancer cell line that endogenously express missense mutant p53^G266E^^29^, and two p53^−/−^ clones were previously generated using CRISPR-Cas9^16^. RT-qPCR and western blotting showed no consistent relationship between *TKT* mRNA or protein expression, respectively, and the presence or absence of mut-p53 protein (Fig. 1A,B). To test the effect of NRF2 activation on TKT expression, H1299 cells were treated with hydrogen peroxide (H_2_O_2_) and mRNA expression analysed by RT-qPCR. Upon H_2_O_2_ treatment and subsequent NRF2 activation (Supplementary Fig. 1A), no change was observed in *TKT* expression in either H1299 p53^null^ or p53^R273H^ cells (Fig. 1C). As a control, expression of *SLC7A11*, a known NRF2 target gene, was also analysed. Consistent with previous findings^16^, *SLC7A11* was strongly upregulated upon H_2_O_2_ treatment in H1299 p53^null^ cells (*p* < 0.001) but not in p53^R273H^ cells (Fig. 1C). In a complementary approach, the effect of depleting NRF2 on *TKT* expression was assessed using siRNA to genetically knock down *NRF2*. *TKT* mRNA expression was found to be unchanged in response to *NRF2* knockdown (Fig. 1D, Supplementary Fig. 1B). In contrast, *SLC7A11* expression was downregulated upon *NRF2* knockdown in p53^null^ cells but not p53^R273H^ H1299 cells, consistent with previous findings^16^. Taken together, these results suggest that TKT expression is not affected by mut-p53 regulation of NRF2 transcriptional activity in H1299 cells.

### Transient knockdown of *TKT* with siRNA slows cell growth and induces cell cycle arrest in H1299 cells independent of mutant-p53 expression

**Figure 2:**
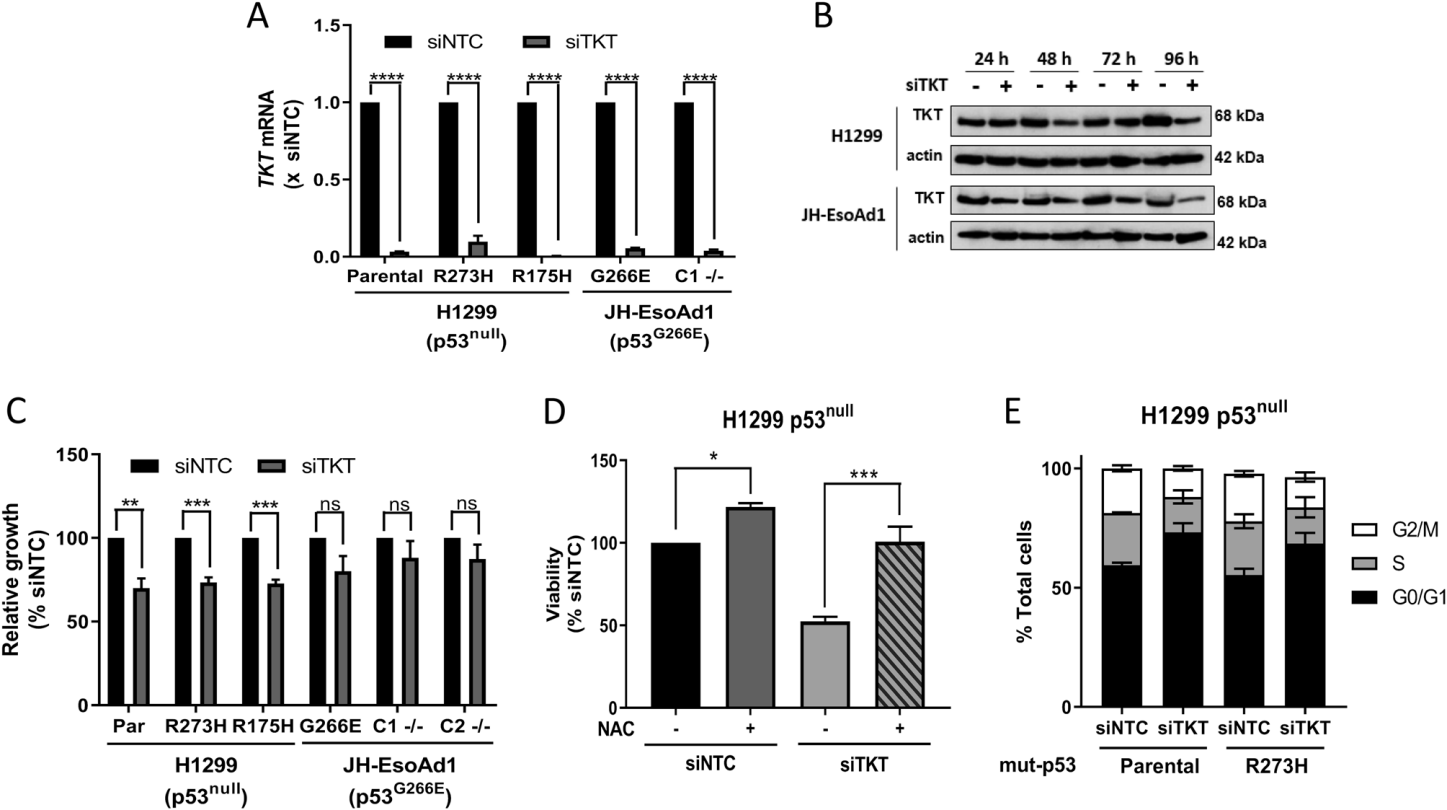
Transient knockdown of TKT slows cell growth and induces cell cycle arrest in H1299 cells irrespective of p53 status. (**A**) *TKT* mRNA expression in H1299 parental (p53^null^), p53^R273H^ and p53^R175H^ cells and JH-EsoAd1 parental (p53^G266E^) and p53−/− clone 1 (C1 −/−) following 24 h TKT knockdown with siRNA, normalised to siNTC (siNTC, non-targeting control; siTKT, *TKT* siRNA). (**B**) Western blots for TKT in H1299 and JH-EsoAd1 parental cells following *TKT* knockdown with siRNA for 24, 48, 72 and 96 h, showing actin as a loading control (**C**) Relative growth of H1299 parental (Par, p53^null^), p53^R273H^ and p53^R175H^ cells and JH-EsoAd1 parental (p53^G266E^), p53−/− clones 1 (C1 −/−) and 2 (C2 −/−) following 96 h *TKT* knockdown with siRNA. (**D**) H1299 parental (p53^null^) cells + /- N-acetyl-cysteine (NAC, 5 mM) −/−) following 96 h TKT knockdown with siRNA. (**C**,**D**) Data are expressed as a percentage of siNTC. (**E**) Changes in cell cycle distribution in H1299 parental (p53^null^) and p53^R273H^ cells following 96 h *TKT* knockdown with siRNA. Data represent mean, error bars = SEM, n = 3 for all studies excluding (**B,E**) where blots and cell cycle analysis are representative of 2 independent experiments. (**A,C**) Unpaired Student’s *t*-test (**D**) One-way ANOVA with Dunnett’s multiple comparison post-test. ns, not significant; *p < 0.05; **p < 0.01; ***p < 0.001; ****p < 0.0001.

Having established that p53 status and NRF2 activation had no apparent bearing on *TKT* expression, we sought to uncover whether cell dependence on TKT varied with respect to p53 status. To examine this, *TKT* was transiently knocked down in H1299 and JHEso-Ad1 cells with the use of siRNA. Despite a highly efficient and rapid knockdown of *TKT* at the mRNA level across all cell lines (Fig. 2A), TKT protein appeared remarkably stable and did not noticeably decline until 96 h after transfection with siRNA (Fig. 2B). This rapid mRNA knockdown with lagging decrease in protein levels was common to all tested cell lines. Depletion of TKT reduced cell proliferation in H1299 cells but not JHEso-Ad1, irrespective of p53 status (Fig. 2C; Supplementary Fig. 2A,B). This diminished cell growth in H1299 cells could be completely rescued by the addition of N-acetyl-cysteine (NAC), a potent antioxidant (Fig. 2D), suggesting that the impact of TKT on cellular redox balance may be tantamount to its effect on cell growth. Because maintaining an optimal redox state is necessary for proper cell replication^30^, the impact of TKT on cell cycle progression with respect to p53 status was also investigated. Knockdown of *TKT* induced arrest in the G0/G1 phase in H1299 cells (Fig. 2E; Supplementary Fig. 2C–F). Moreover, this effect was seen in both H1299 p53^null^ and p53^R273H^ cell lines. Altogether, these data support the notion that the role of TKT in moderating oxidative stress may be required for cell proliferation, p53 status notwithstanding, but is cell context dependent.

### Transient knockdown of *TKT* with siRNA sensitises p53^null^ cells to APR-246

**Figure 3:**
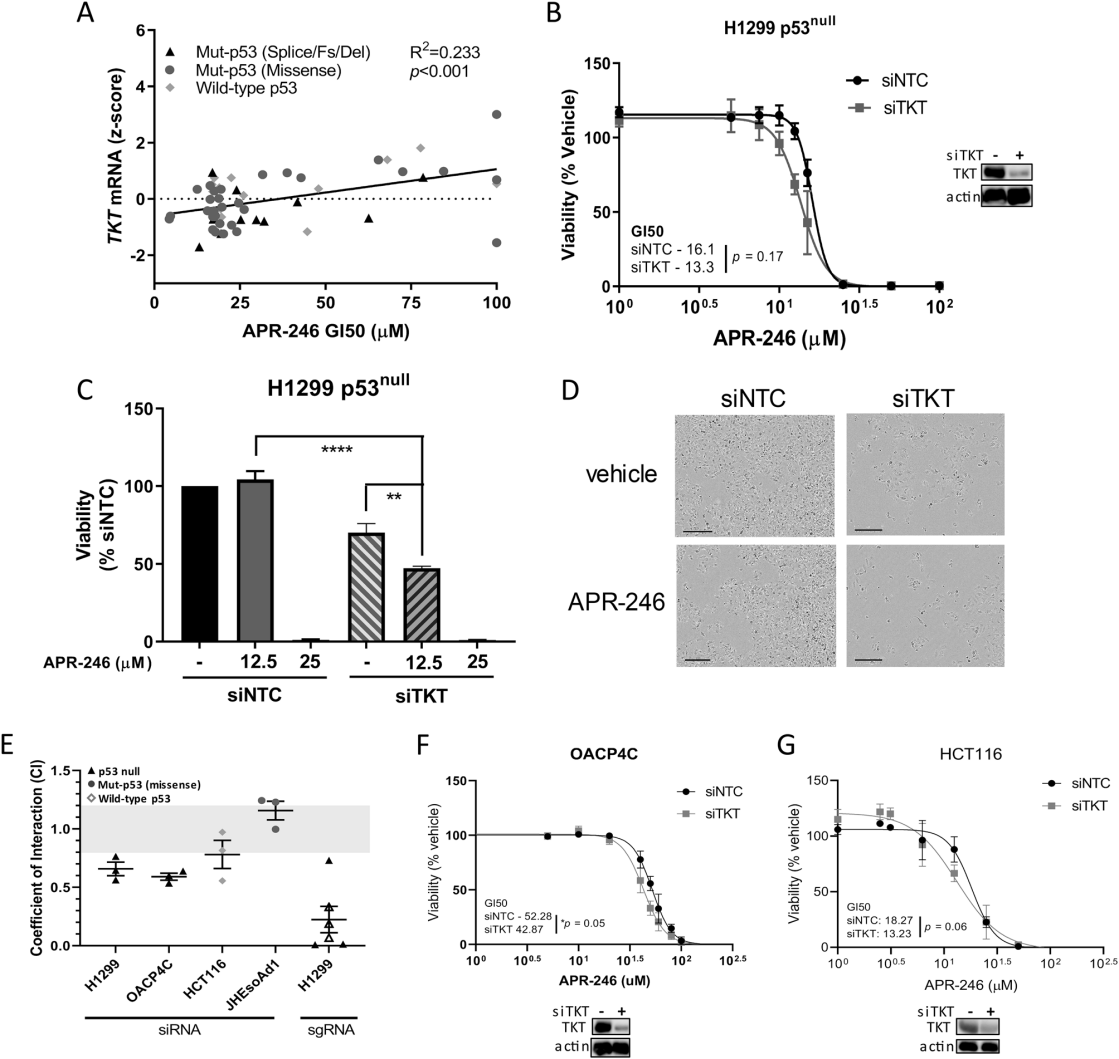
Acute knockdown of *TKT* sensitises p53^null^ cells to APR-246. (**A**) Correlation between *TKT* mRNA levels and APR-246 GI50 (50% growth inhibitory concentration) across the NCI-60 cancer cell line panel^31^. Each point represents an individual cell line. The NCI-60 is a panel of 60 diverse human cancer cell lines used by the National Cancer Institute for therapeutic development^32^. (**B**) (left) Cell viability (AlamarBlue) in H1299 **c**ells following 72 h treatment with increasing concentrations of APR-246, administered 24 h after *TKT* knockdown with siRNA, normalised to vehicle control (siNTC, non-targeting control; siTKT, *TKT* siRNA (right) Western blots for TKT in H1299 cells following *TKT* knockdown with siRNA for 96 h, showing actin as a loading control. (**C**) Cell viability (AlamarBlue) in H1299 parental (p53^null^) cells following 72 h APR-246 treatment (12.5 or 25 µM), administered after 24 h *TKT* knockdown with siRNA, normalised to siNTC vehicle control. (**D**) Digital microscope images (Incucyte FLR) depicting cell death following 72 h treatment with APR-246 (15 µM), administered 24 h after *TKT* knockdown with siRNA (scale bars = 100 µm). (**E**) Interaction between *TKT* knockdown and APR-246 in H1299, OACP4C, HCT116 and JH-EsoAd1 cells based on the Bliss Independence model. Data represent most synergistic interaction across APR-246 dose response. A coefficient of interaction < 0.8 indicates synergy, 0.8–1.2 indicates additivity and > 1.2 indicates antagonism. Cell viability (AlamarBlue) in (**F**) OACP4C and (**G**) HCT116 cells following 72 h treatment with increasing concentrations of APR-246, administered 24 h after *TKT* knockdown with siRNA, normalised to vehicle control (bottom) Western blots for TKT following 96 h knockdown with siTKT, showing actin as a loading control (siNTC, non-targeting control; siTKT, *TKT* siRNA. Data represent mean, error bars = SEM, n = 3 for all studies. (**C**) One-way ANOVA with Dunnett’s multiple comparison post-test. ns, not significant; *p < 0.05; **p < 0.01; ***p < 0.001; ****p < 0.0001.

We next investigated the relationship between TKT and the antioxidant-depleting drug APR-246. Analysis of the NCI-60 human tumour cell line screen found that *TKT* expression correlated with sensitivity to PRIMA-1, an unmethylated analogue of APR-246 (Fig. 3A)^31,32^. Increased expression of *TKT* mRNA was positively correlated with an increased growth inhibitory concentration (GI50) of APR-246. This suggests a potential protective effect of TKT against APR-246.

Transient knockdown of *TKT* did not alter the response of cells expressing mutant p53 protein, nor when the endogenous mutant *TP53* was knocked out in JH-EsoAd1 (Supplementary Fig. 3A–E). Interestingly, we noticed that knockdown of *TKT* increased sensitivity of H1299 p53^null^ cells to low doses of APR-246, with a coefficient of interaction (CI) of 0.66 ± 0.06 indicative of synergistic activity (Fig. 3B–D, Supplementary Fig. 3F). Synergistic interactions were also observed in Cas9-expressing H1299 p53^null^ cells transduced with two independent synthetic guide RNAs (sgRNAs) targeting *TKT* (Fig. 3E, Supplementary Fig. 3F). To investigate this further, we utilised another cell line with an endogenous *TP53* mutation that results in a p53 null status (OACP4C, Fig. 3F) and HCT116 cells (Fig. 3G), which express wild-type p53 protein. Similar to H1299 p53^null^ cells, OACP4C p53^null^ cells showed synergistic activity between *TKT* knockdown and APR-246 (Supplementary Fig. 3A), as well as a significant decrease in the GI50 of APR-246 in TKT depleted cells. In contrast, the maximal CI in HCT116 cells was 0.78 ± 0.12 (Supplementary Fig. 3A) indicative of an additive/borderline-synergistic effect. Thus, knockdown of *TKT* is synergistic with APR-246 in cancer cell lines with an endogenous p53 null phenotype.

### Transient knockdown of *TKT* augments oxidative stress levels, resulting in increased cell death

**Figure 4:**
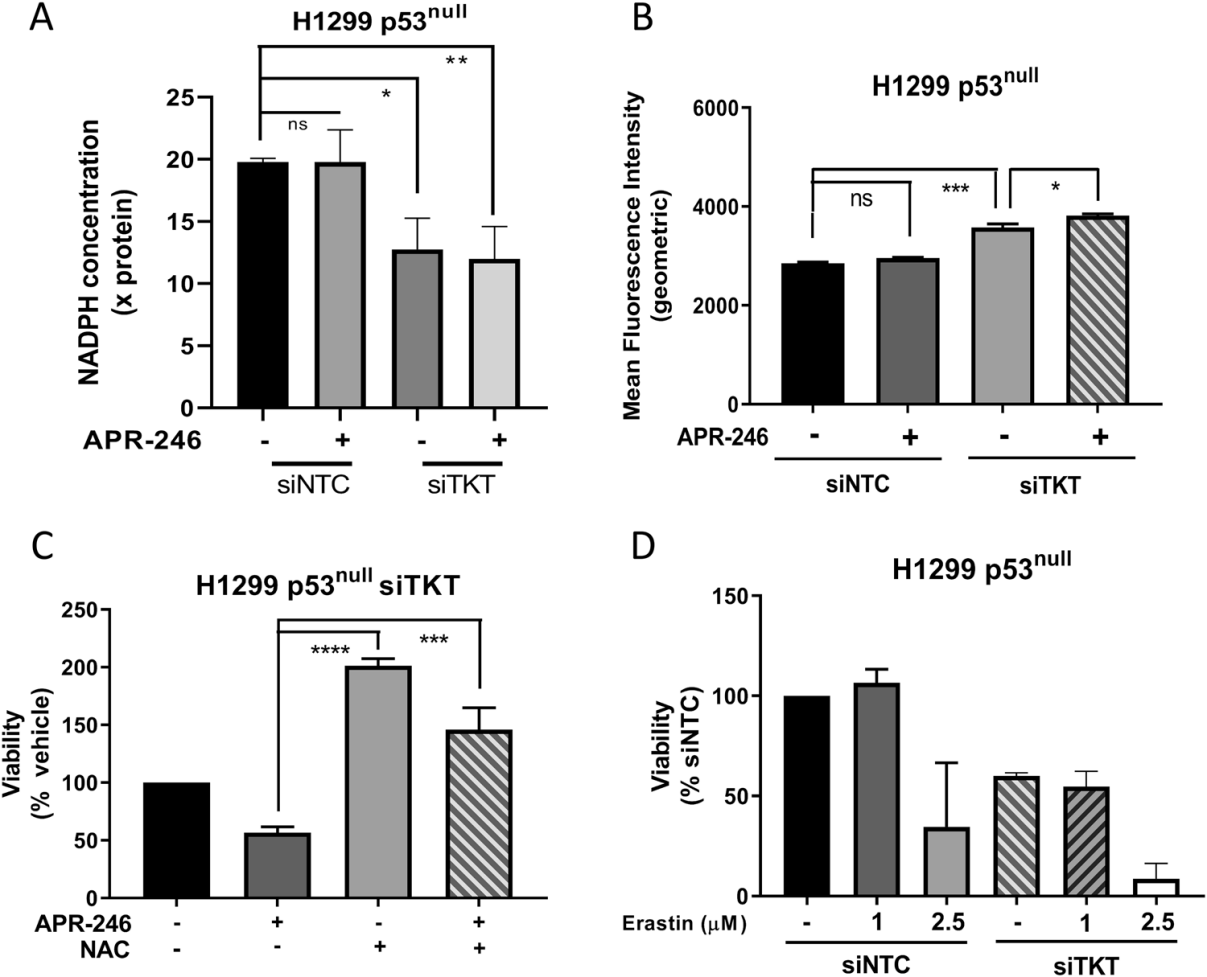
Transient knockdown of *TKT* with siRNA augments oxidative stress levels, but does not increase sensitivity to Erastin in H1299 p53^null^ cells. (**A**) Concentration of NADPH, normalised to total protein concentration, in H1299 parental (p53^null^) cells following 18 h APR-246 (25 µM) treatment administered after 72 h *TKT* knockdown with siRNA (siNTC, non-targeting control; siTKT, *TKT* siRNA). (**B**) Geometric mean fluorescence intensity of MitoSOX Red in H1299 parental (p53^null^) cells following 18 h APR-246 (25 µM) treatment administered after 72 h *TKT* knockdown with siRNA, measured with flow cytometry (siNTC, non-targeting control; siTKT, *TKT* siRNA). (**C**) Cell viability in H1299 parental (p53^null^) cells following 72 h APR-246 (15 µM) treatment and/or N-acetyl-cysteine (NAC, 5 mM) supplementation, following 24 h TKT knockdown with siRNA, shown as a percentage of vehicle control. (**D**) Cell viability (AlamarBlue) in H1299 **c**ells following 72 h treatment with 1 or 2.5 µM of Erastin, administered 24 h after *TKT* knockdown with siRNA, normalised to siNTC vehicle control (siNTC, non-targeting control; siTKT, *TKT* siRNA). Data represent mean, error bars = SEM, n = 3 for all studies. (**A,B,C**) One-way ANOVA with Dunnett’s multiple comparison post-test. ns, not significant; *p < 0.05; **p < 0.01; ***p < 0.001; ****p < 0.0001.

We next investigated whether the sensitisation of H1299 p53^null^ cells to APR-246 through *TKT* knockdown was a product of the combined effect of these conditions on oxidative stress levels. Treatment with APR-246 (25 µM) for a relatively short period (18 h) did not significantly alter the levels of NADPH (Fig. 4A) nor mitochondrial superoxides in H1299 cells (Fig. 4B; Supplementary Fig. 3G). In contrast, *TKT* knockdown significantly decreased NADPH levels (Fig. 4A) and increased mitochondrial ROS in H1299 p53^null^ cells, which was further augmented by APR-246 (*p* < 0.05) (Fig. 4B; Supplementary Fig. 3G). Treatment with NAC entirely rescued the combination effect of *TKT* knockdown and APR-246 on cell viability (*p* < 0.001) (Fig. 4C). Altogether, these results indicate that the combination of *TKT* knockdown and APR-246 treatment induces a greater oxidative stress environment than either condition alone. The fact that the sensitisation of H1299 p53^null^ cells to APR-246 by *TKT* knockdown was rescued by antioxidant treatment suggests that greater intracellular ROS levels are the cause of the increased cell death. However, transient knockdown of *TKT* did not sensitise H1299 cells to Erastin, a known inducer of oxidative stress (Fig. 4D, Supplementary Fig. 3I), suggesting a more complex mechanism at play in the context of APR-246.

### Overexpression of *TKT* reduces sensitivity to APR-246 in H1299 p53^null^ cells

**Figure 5:**
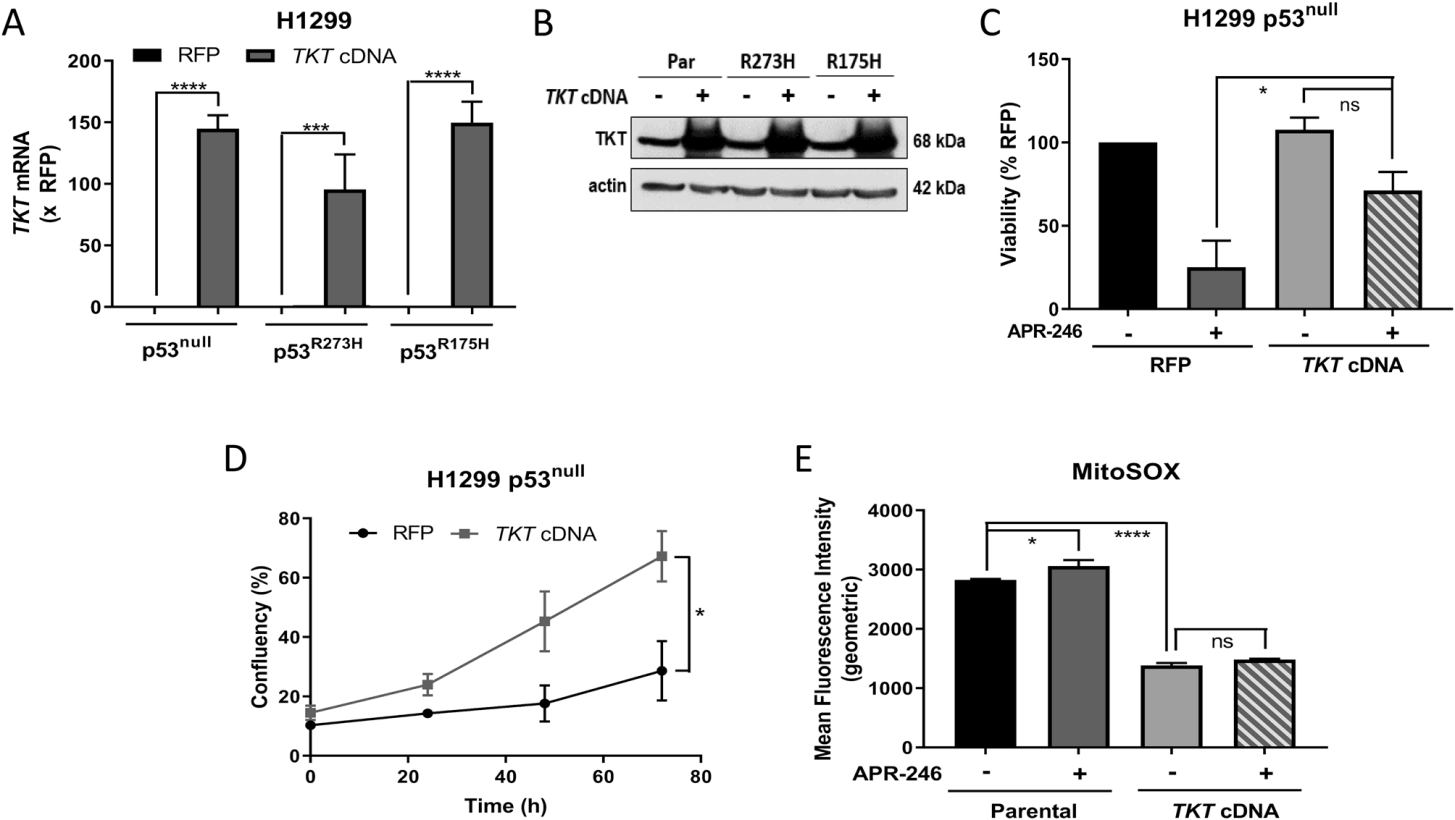
Overexpression of TKT induces resistance to APR-246 in p53-null cells and protects against APR-246-induced superoxides. (**A**) *TKT* mRNA expression and (**B**) western blots for TKT in H1299 parental (p53^null^), p53^R273H^ and p53^R175H^ cells with either *TKT* or red fluorescent protein (RFP) overexpression. (**A**) Expression normalised to RFP, (**B**) actin is shown as loading control. (**C**) Cell viability and (**D**) proliferation of H1299 parental (p53^null^) cells with either *TKT* or red fluorescent protein (RFP) overexpression following 72 h APR-246 treatment (15 µM). (**E**) Geometric mean fluorescence intensity of MitoSOX Red in H1299 parental (p53^null^) cells either untransfected (Parental) or overexpressing *TKT* (*TKT* cDNA) with 18 h APR-246 treatment (25 µM) measured with flow cytometry. Data represent mean, error bars = SEM, n = 3 for all studies excluding (**B**) where blots are representative of 2 independent experiments. (**A**) Unpaired Student’s *t*-test on raw data. (**C**,**E**) One-way ANOVA with Dunnett’s multiple comparison post-test. (**D**) Unpaired Student’s *t*-test. ns, not significant; *p < 0.05, ***p < 0.001, ****p < 0.0001.

We next determined the effect of overexpressing *TKT* on response to APR-246 in H1299 cells. Consistent with the effect of transient *TKT* knockdown, overexpression of *TKT* (Fig. 5A,B) conferred resistance to APR-246 in H1299 p53^null^ cells (Fig. 5C,D; Supplementary Fig. 4A) but this effect was not seen in H1299 p53^R273H^ (Supplementary Fig. 4B) or p53^R175H^ cells (Supplementary Fig. 4C). Consistent with this, overexpression of *TKT* significantly reduced basal mitochondrial ROS and protected against APR-246-induced superoxides in H1299 p53^null^ cells (Fig. 5E; Supplementary Fig. 4D). However, the protective effect of *TKT* overexpression in these cells did not extend to long-term survival as assessed in clonogenic assays (Supplementary Fig. 4E). Combined, these data support the notion that overexpression of *TKT* delays the onset of APR-246-induced cell death in H1299 p53^null^ cells.

### Inhibition of NADPH production slows cell growth in H1299 p53^null^ cells but does not affect sensitivity to APR-246 or induce cell cycle arrest

**Figure 6:**
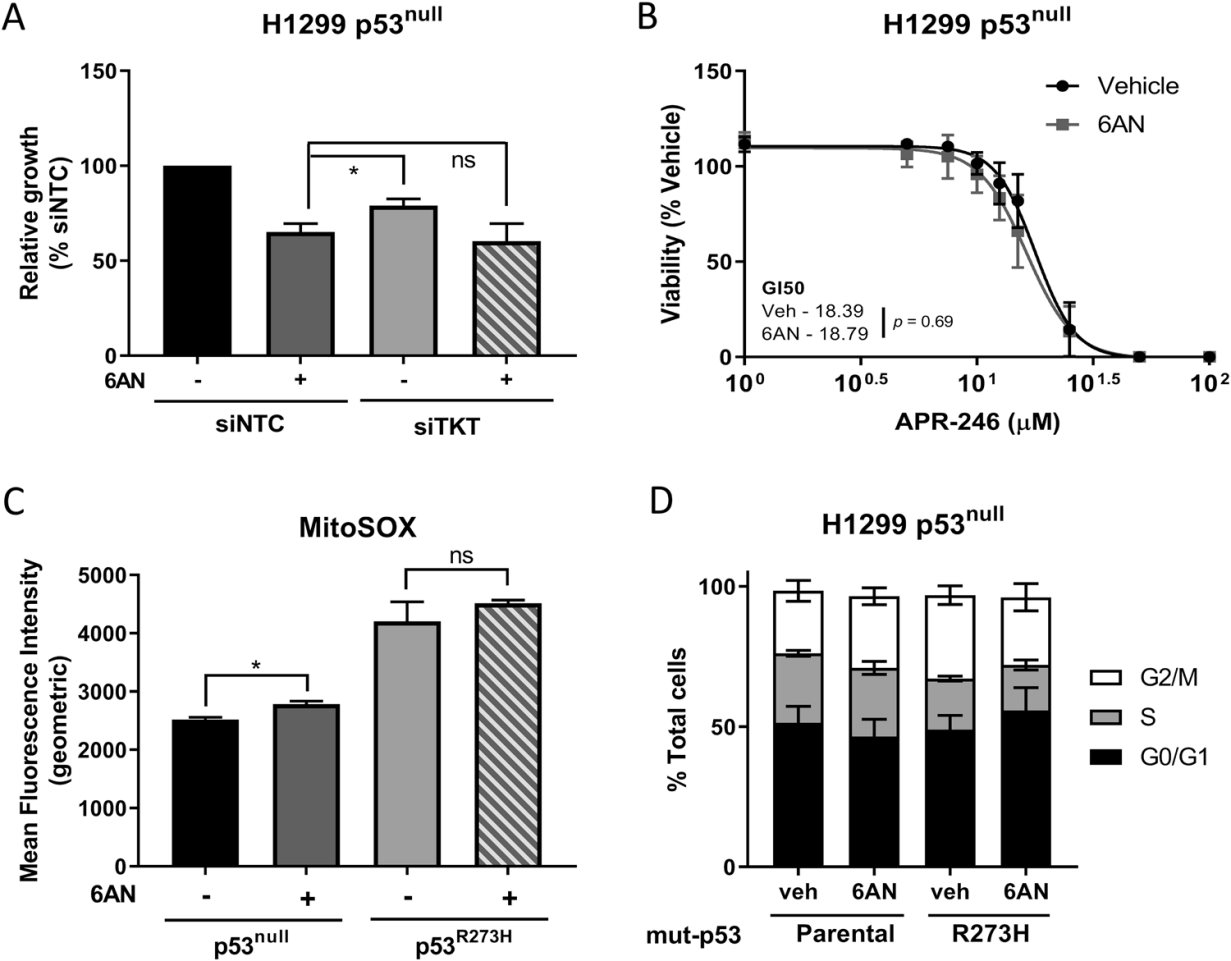
Inhibition of NADPH production slows cell growth but does not sensitise p53^null^ cells to APR-246 or induce cell cycle arres. (**A**) Cell viability in H1299 parental (p53^null^) cells following *TKT* knockdown with siRNA or transfection with non-targeting control in combination with 72 h 6-aminonicotinamide (6AN; 10 µM) treatment, normalised to non-targeting control (siNTC, non-targeting control; siTKT, *TKT* knockdown with siRNA). (**B**) Cell viability in H1299 parental (p53^null^) cells following 72 h 6AN (10 µM) or vehicle treatment in combination with increasing doses of APR-246. (**C**) Geometric mean fluorescence of MitoSOX Red in H1299 parental (p53^null^) and p53^R273H^ cells following 72 h treatment with 6AN (10 µM) or vehicle measured by flow cytometry. (**D**) Changes in cell cycle distribution in (left) H1299 parental (p53^null^) and (right) p53^R273H^ cells following 24 h treatment with 6AN (veh, vehicle control). Data represent mean, error bars = SEM, n = 3 for all studies. (**A**) One-way ANOVA with Dunnett’s multiple comparison post-test. (**C**) Unpaired Student’s t-test. ns, not significant; *p < 0.05.

A major role of TKT in the PPP is the shuttling of intermediates back into glycolysis, from where they return to the oxidative arm of the PPP and contribute to the production of NADPH^33^. Given this, we next investigated whether treatment with 6-aminonicotinamide (6AN), an inhibitor of NADPH production (Supplementary Fig. 5A), would elicit the same effects as *TKT* knockdown. 6AN treatment elicited a significantly greater reduction in growth rate than that caused by *TKT* knockdown, but this effect was not enhanced when *TKT* knockdown and 6AN treatment were administered concomitantly (Fig. 6A). Further, 6AN treatment did not sensitise H1299 p53^null^ cells to APR-246 (Fig. 6B), and no significant shift in the GI50 of APR-246 was observed in these cells (*p* = 0.14), despite significantly increased mitochondrial superoxides following treatment with 6AN, indicating drug activity (Fig. 6C; Supplementary Fig. 5B). The impact of 6AN on cell cycle progression was also assessed. After 24 h of treatment with 6AN, neither H1299 p53^null^ nor p53^R273H^ cells showed any sign of cell cycle arrest (Fig. 6D; Supplementary Fig. 5C–F). This is in stark contrast to the clear interference with cell cycling caused by *TKT* knockdown. Taken together, these results suggest some similarities between the consequences of *TKT* knockdown and inhibition of NADPH production by 6AN, but 6AN treatment does not entirely phenocopy the effects of *TKT* gene knockdown.

## Discussion

Despite the clear parallels between these factors relating to antioxidant response mechanisms, the relationship between mut-p53 and the role of the PPP and TKT in the antioxidant response remains poorly understood. This work aimed to delineate the relationship between TKT and mut-p53, as well as TKT and the drug APR-246, which has both mutant-p53 reactivating and oxidative stress-inducing mechanisms. Using isogenic models of p53^null^ or mut-p53 cells and additional cell lines with varying p53 status, we examined the effects of perturbations to TKT on oxidative stress and sensitivity to APR-246, relative to p53 status. Our findings indicate a potential role for TKT in modulating sensitivity to APR-246, but suggest that this is likely not via altering redox balance.

Previous studies dictate that accumulated mut-p53 inhibits the transcriptional ability of NRF2^16,19,20^ whilst NRF2 has been found to transcriptionally activate *TKT*^25^. From this, it may be inferred that through inhibition of NRF2, accumulated mut-p53 may impact transcription of *TKT*. In contrast, our work determined that neither manipulation of mut-p53 nor NRF2 had any bearing on expression of *TKT* in H1299 or JH-EsoAd1 cells (Fig. 1A–D). This suggests that mechanisms of *TKT* regulation may differ among cancer cell types or those with altered dependency on NRF2. For example, *TKT* was found to be a transcriptional target of NRF2 in A549 cells which harbour endogenous KEAP1 mutation, leading to constitutively active NRF2 in these cells^26^. Given that neither cell line used in this study contain KEAP1 mutations, is it possible that this difference may account for the discrepancy between our work and previous findings.

Consistent with previous findings^25^, we found that *TKT* depletion hinders cell growth by arresting the cell cycle, and that this occurred irrespective of p53 status (Fig. 2C,E). That this could be rescued with antioxidant treatment (i. e. NAC; Fig. 2D) is in congruence with previous studies involving TKT and other PPP enzymes, such as ribose-5-phosphate isomerase (RPIA)^34^. RPIA converts ribulose-5-phosphate (Ru5P) into ribose-5-phosphate (R5P) in the PPP and thus plays a major role in ribonucleotide biosynthesis^35^. This similarity strongly suggests an ability of NAC to counteract the decline in ribonucleotide synthesis that is likely caused by depletion of these PPP enzymes.

Although these early data did not identify any relationship between TKT and mut-p53, it remains that, according to NCI-60 data, a correlation exists between *TKT* expression and sensitivity to PRIMA-1 (Fig. 3A). Critically, we found that transient knockdown or knockout of *TKT* sensitised only p53^null^ cells, and not cells expressing mut-p53, to low doses of APR-246 (Fig. 3B–F, Supplementary Fig. 3A–F), whilst overexpression of *TKT* conferred resistance to the drug, again only in p53^null^ cells (Fig. 5C). Notably, in contrast to cells with an endogenous p53 null mutation, *TKT* knockdown did not increase sensitivity in JH-EsoAd1 cells in which the endogenous missense mut-p53 had been knocked out using CRISPR/Cas9. While the reason for this remains unclear, we speculate that this may reflect differences between tumour cells that have evolved without the ability to express any p53 protein (H1299 and OACP4C cells) compared to cells that evolved expressing a missense mut-p53 that was then knocked out.

Our results in mut-p53 expressing cells were surprising, given previous findings that mut-p53 expressing cells are highly sensitive to further assaults on cellular redox balance (particularly the antioxidant-depleting effects of APR-246) due to inherent impaired antioxidant function^11,16^. However, mut-p53 has been shown to transactivate a range of anti-apoptotic and growth enhancing factors^8,9,36,37^ as well as alter tumour cell metabolism to promote survival^7,38,39^. In fact, it has been found that the antioxidant thioredoxin is upregulated in mut-p53 expressing cells^11^, which alludes to a thioredoxin-dependent mechanism that allows mut-53 cells to compensate for APR-246-induced glutathione depletion. As such, it is possible that the mut-p53 expressing cells were unaffected by the consequences of *TKT* knockdown due to upregulation of pro-survival measures or prioritisation of alternative metabolic pathways.

Upon further investigation of oxidative balance, and consistent with the findings of Xu et al.^25^, we found that in all cell types, knockdown (Fig. 4A,B) and overexpression (Fig. 5E) of *TKT* increased and decreased intracellular ROS burden, respectively. These data suggest that modulations to *TKT* influence flux through the oxidative arm of the PPP and subsequent production of NADPH. Intriguingly, this occurred in both p53^null^ and mut-p53 expressing cells, leading to the conundrum of why *TKT* depletion only induced sensitivity to APR-246 in p53^null^ cells, whilst the effect on ROS levels was also seen in mut-p53 expressing cells. Cancer cells natively exhibit higher levels of oxidative stress than untransformed cells^30,40,41^. Basal ROS levels in mut-p53 expressing cells were found to be higher still, in comparison to p53^null^ cells (Supplementary Fig. 3H; compare with Fig. 4B), congruent with previous findings^16,21^, likely due to the suppressing effect of mut-p53 on NRF2-dependent antioxidant pathways. Despite higher levels of oxidative stress, mut-p53 cancers have been found to show improved survival and augmented proliferation in response to oxidative stress^21^. Mut-p53 is also thought to be able to commandeer additional pro-survival pathways^8,9,36,37^. Thus, it is possible that due to these gains-of-function, mut-p53 expressing cells were able to escape the effects of *TKT* knockdown that increased APR-246-induced cell death in p53^null^ cells.

TKT has a dualistic role in the PPP and its activity modulates depending on the metabolic needs of the cell^33^. It accomplishes this by either shuttling intermediates back towards the oxidative arm of the PPP to produce NADPH in response to oxidative demand, or producing R5P to promote production of ribonucleotides^33^. In order to separate these effects and their potential role in altering sensitivity to APR-246, 6-aminonicotinamide (6AN), a compound that impedes NADPH production but is not known to effect production of ribonucleotides, was utilised. 6AN inhibits the two major NADPH-producers in the PPP, 6-phosphogluconoate (6PGD) and glucose-6-phosphate dehydrogenase (G6PD)^42,43^. In this way, the isolated effect of NADPH depletion on APR-246 sensitivity was able to be investigated, without the alternative function of TKT (i. e. ribonucleotide production) confounding the results. Intriguingly, 6AN slowed cell growth (Fig. 6A) despite eliciting no effect on cell cycle progression (Fig. 6D), irrespective of p53 status. In contrast to transient *TKT* knockdown, 6AN treatment did not significantly alter sensitivity to APR-246 in H1299 p53^null^ cells (Fig. 6B), despite eliciting a similar effect on intracellular ROS levels (Fig. 6C). This suggests that impaired R5P production may be the driving force behind *TKT* knockdown-induced APR-246 sensitivity in p53^null^ cells, rather than increased levels of ROS due to inadequate antioxidant mechanisms. Additionally, *TKT* knockdown did not increase sensitivity to erastin, a known oxidative stress inducer, indicating that loss of TKT expression is not broadly sensitising cells to oxidative stress-mediated cell death. Imperatively, this suggests that the mechanism of action of APR-246 is more complex than previously described and may incorporate aspects of macromolecule biosynthesis that have not been explored previously in this context. Future work is therefore required to examine the effect of TKT knockdown on R5P production and the impact of this on APR-246 sensitivity and how this is related to p53 status.

Despite similar TKT expression levels across cells in this model with varying p53 status, a distinct change in sensitivity to APR-246 was observed when *TKT* was ectopically overexpressed or transiently depleted in p53^null^ cells. This, combined with correlative data from the NCI-60 human tumour cell line screen (Fig. 3A), suggests that TKT expression regulates sensitivity to APR-246, at least in p53^null^ tumours. Of particular interest is the evidence that sensitivity of mut-p53 cells to APR-246 is not regulated in this same way in this model. We propose a model to explain this observation based on the induction of wild-type-p53-like function in mut-p53 cells when treated with APR-246 (Supplementary Fig. 6). Wild-type-p53 has been found to inhibit activity of G6PD, the rate-limiting enzyme of the PPP, upstream of TKT, by preventing dimerization of the enzyme^44^. Because treatment with APR-246 induces wild-type-p53-like conformation and activity in mut-p53 cells, mut-p53 cells treated with APR-246 sustain impeded function of the PPP via G6PD inhibition. Therefore, due to upstream interference and consequential limited flux through the PPP, modulations of downstream TKT may have no effect on APR-246 activity. Treatment of p53^null^ cells with APR-246 would not confer this same wild-type-p53-like effect. Thus, modulations of TKT in p53^null^ cells would strongly interfere with the previously intact PPP, rendering the cells more susceptible to APR-246 treatment. This is consistent with our finding that upstream interference with the PPP via G6PD inhibition by 6AN had no synergistic or additive effect in combination with TKT modification in any cell lines, irrespective of p53 status. In summary, this study bridges the gaps in current knowledge surrounding the relationship between the PPP, mut-p53 cancers and therapies. The findings indicate that whilst mut-p53 is known to interfere with NRF2, it did not affect *TKT* in the investigated models. Furthermore, it is proposed that regulation of *TKT* is not as simple as per the previously suggested mechanism of NRF2 activation^25^, and that other factors must certainly be at play. This study also addressed the growing complexity of mut-p53 gain-of-function capabilities. Given the failure of NRF2 modulation to affect TKT expression in this study, future research is needed to explore the role of mut-p53 in regulating the antioxidant response and its influence on the PPP. The present findings also mar the current predilection in research for targeting cellular redox balance as an avenue for chemotherapeutics, particularly for mut-p53 cancers. This is due, in part, to growing evidence of the complex nature of mut-p53 in the antioxidant response, as well as the uncovering of the potential influence of external factors. Finally, this study has identified *TKT* as a determinant for APR-246 sensitivity in p53^null^ cells, which may open a new avenue for exploration and potentially allow for personalised, effective treatment for p53^null^ cancers.

## Materials and methods

### Cell culture

HEK293T (RRID:CVCL_0063) cells were purchased from American Tissue Culture Collection. NCI-H1299 (RRID:CVCL_0060) and mutant p53 expressing sublines were a kind gift from Prof. Ygal Haupt (Peter MacCallum Cancer Centre, Melbourne, Australia). JH-EsoAd1 (RRID:CVCL_8098) cells were obtained from Professor James Eshleman (Johns Hopkins University, Baltimore, Maryland, USA). OACP4C (RRID:CVCL_1843) cells were obtained from Rebecca Fitzgerald (University of Cambridge, UK). HCT116 (RRID:CVCL_0291) cells were obtained from Karen Sheppard (Peter MacCallum Cancer Centre, Australia). H1299, JH-EsoAd1 and OACP4C cells were cultured in RPMI 1640 containing 2.5 mM L-glutamine (Gibco, ref 11875-093), HCT116 and HEK293T cells were cultured in DMEM (Gibco, ref 11965-092) and all media supplemented with 10% heat inactivated foetal bovine serum (FBS) and 1% penicillin and streptomycin (Gibco, ref 15140-122) unless otherwise stated. All cells were incubated at 37 °C and 5% CO_2,_ STR genotyped to confirm identity and regularly tested for mycoplasma contamination. Unless otherwise indicated, seeding densities were as follows for H1299 and JH-EsoAd1 cells: 2 × 10^3^ in 96-well format, 4 × 10^5^ for less than 24 h incubation and 2 × 10^5^ for incubation times between 24 and 48 h in 6-well format and 6 cm dishes. OACP4C cells were seeded at 1 × 10^4^ and HCT116 at 3 × 10^3^ in 96-well format.

### Transient TKT and NRF2 knockdown

Cells were reverse transfected with 40 nM NRF2 (siGenome Smartpool, Dharmacon), TKT or non-targeting control siRNA pools (ON-TARGETplus SMARTpool, Dharmacon) using Lipofectamine RNAiMax solution (Life Technologies) as per manufacturer’s guidelines. Knockdown efficiency was assessed by reverse transcription quantitative polymerase chain reaction (RT-qPCR) and western blotting. siRNA sequences are detailed in Supplementary Table 1.

### TKT knockout using CRISPR/Cas9

H1299 cells expressing constitutive Cas9 endonuclease (FUCas9Cherry, kind gift of Marco Herold, WEHI, Australia) were reverse transfected with two independent TKT sgRNA (Integrated DNA Technologies, IDT) at 30 nM using Lipofectamine RNAiMax solution (Life Technologies) according to the manufacturer’s protocol. Knockout efficiency was assessed by western blotting.

### Overexpression of TKT

TKT was ectopically overexpressed in H1299 cells using Precision LentiORF TKT with stop codon (clone ID: PLOHS_100005708) or red fluorescent protein (RFP) as a control. Viral particles were produced by transfecting HEK293T cells using the Lenti-X Packaging System (Clontech Laboratories) as per manufacturer’s directions. Subsequently, target cell lines were transduced with viral supernatants in the presence of 8 ng/µL polybrene. Transduced cells were collected via cytometric sorting (BD Fusion3, BD Bioscience). Expression was confirmed by RT-qPCR and western blotting.

### Gene expression using RT-qPCR

Cells were seeded in 6 cm dishes and allowed to adhere overnight. Following the relevant experiments, extraction and purification of RNA was conducted using Nucleospin RNA kit (Macherey–Nagel) before reverse transcription to cDNA using Transcriptor First Strand cDNA Synthesis Kit (Roche) as per manufacturer’s instructions, including the optional denaturing step. Quantitative PCR (qPCR) was conducted using LightCycler 480 SYBR-Green qPCR (Roche) as per manufacturer’s protocol, with gene expression normalised within each sample to GAPDH and analysed using the ΔΔCt method. Primer sequences were obtained using the NCBI Primer Blast application and are detailed in Supplementary Table 2. Product sizes were confirmed via electrophoresis on 1% agarose gels with size comparison to Hyperladder 100 bp (Bioline).

### Western blotting

Cells were seeded in 6 cm dishes and allowed to adhere overnight. Following the relevant experiments, cells were lysed on ice in RIPA buffer (1 mM EDTA; 1% v/v NP40; 0.5% w/v sodium deoxycholate; 0.1% v/v SDS; 50 mM sodium fluoride; 1 mM sodium pyrophosphate in PBS, SigmaAldrich) containing phosphatase (PhosphoSTOP, Roche) and protease (Complete ULTRA, Roche) inhibitors. Protein concentrations were quantified using the DC Protein assay (Bio-Rad). Equivalent amounts of protein lysates were boiled, resolved by SDS-PAGE using 10% w/v acrylamide gels, and transferred to polyvinylidene difluoride membranes. Membranes were incubated for 1 h in blocking buffer (5% w/v skim milk, 0.05% v/v Tween-20 in Tris-buffered saline; TBS-T) and probed overnight at 4 °C with the primary antibody. Blots were washed thrice in wash buffer (0.05% v/v TBS-T) for 10 min, followed by incubation with peroxidase-conjugated secondary antibody (Dako) for 1 h at room temperature. Proteins were visualised by Amersham ECL western Blotting Detection Reagents (GE Life Sciences) or ECL Plus western blotting substrate kit (Thermo Scientific). Anti-β-actin or anti-GAPDH antibodies were used to assess protein loading. Antibodies are detailed in Supplementary Table 3.

### Cell cycle analysis

Cells were seeded in 24-well plates and exposed to siRNA TKT knockdown for 96 h, 6-aminonicotinamide (6AN) for 72 h or vehicle prior to harvesting. Cells were washed with PBS, trypsinised, pelleted by centrifugation at 2400 rpm for 5 min and the supernatant removed before resuspension in PBS supplemented with 1% v/v FBS and fixing in ice cold 100% ethanol. Cells were then pelleted again and washed twice with PBS/1% v/v FBS prior to staining for 90 min at room temperature in the dark with 25 µg/mL propidium iodide (Sigma-Aldrich) and 40 µg/mL RNAse A in PBS/1% v/v FBS. At least 1 × 10^4^ single cell events were recorded by flow cytometry (BD FACSCanto II, BD Bioscience) and analyzed using Flowlogic software (Inivai Technologies). The proportion of cells in each phase of the cell cycle was expressed as a percentage of total cell population.

### NADPH measurement

Total NADPH was measured using a NADPH Assay Kit (Abcam) as per the manufacturer’s instructions. Cells (1 × 10^5^ per well) were seeded in 6-well plates and exposed to siRNA TKT knockdown for 96 h or 24 h before treatment with APR-246 (25 μM) for 18 h or 6AN (10 μM) for 72 h respectively. Cells were washed with PBS, harvested by scraping in lysis buffer and centrifuged. Supernatant was extracted and absorbance at 460 nm was measured using a Cytation 3 microplate reader (Molecular Devices). Total NADPH concentration was calculated from an internal standard curve and normalised to protein concentration.

### ROS detection

MitoSOX Red reagent (ThermoFisher Scientific) was utilised to detect mitochondrial-specific ROS accumulation, using DAPI as a viability marker counter-stain. H1299 cells were seeded in 24-well plates and exposed to siRNA TKT knockdown for 78 h before treatment with APR-246 (25 µM) 18 h prior to ROS detection. Complete media containing 5 µM MitoSOX Red reagent was applied to cells and incubated at 37 °C for 30 min prior to analysis. Media was removed and replaced with media containing 1 µg/mL DAPI and incubated in the dark for 15 min at room temperature. Cells were then washed three times, trypsinised and resuspended in PBS supplemented with 1% v/v FBS and 25 mM EDTA before ROS-induced fluorescence was quantified using the FACSymphony (BD Biosciences).

### Cellular proliferation and viability assays

Cellular proliferation was assessed using a microscopy-based live cell imaging system (Incucyte FLR, Essen BioScience). Cells were seeded in 96-well plates and treated with indicated doses of APR-246 or vehicle for 72 h. Cells were imaged every 24 h. The confluency of APR-246 treated wells was normalised to pre-treatment reading and compared with vehicle treatment. To assess cell viability, AlamarBlue (Life Technologies) fluorometric assays were conducted as per established protocols^45^. Following treatments, 20 µL of 2% v/v AlamarBlue was added to each well and incubated for 2 h at 37 °C. Fluorescence was measured using a FLUOstar OPTIMA microplate reader (BMG Labtech) at an excitation of 540 nm and an emission of 590 nm. Results were normalised to vehicle-treated wells.

### APR-246 dose responses, antioxidant rescue and NAPDH inhibition

Cells were seeded at 2 × 10^3^ cells/well in 96-well plates, exposed to siRNA TKT knockdown or non-targeting control and allowed to adhere overnight. The following day, media was replaced with fresh media supplemented with APR-246 or vehicle control. To determine GI50 values, cells were exposed to a range of concentrations of APR-246 (1 to 100 µM) for 72 h and assayed using AlamarBlue. For antioxidant rescue, after 24 h of siRNA TKT knockdown, cells were treated with 5 mM N-acetylcysteine (NAC) or 15 μM APR-246, individually or in combination, for 72 h before assaying with AlamarBlue. For NADPH inhibition, after 24 h of siRNA TKT knockdown, cells were treated with 10 µM 6-aminonicotinamide (6-AN) or vehicle control in combination with a range of APR-246 concentrations for 72 h before assaying with AlamarBlue. APR-246 dose responses both with and without NADPH inhibition were repeated in cells overexpressing TKT or RFP control, in the aforementioned manner, without exposure to siRNA.

### Colony formation assay

Cells in six-well plates were treated with 50 µM APR-246 for 5, 10, 15, 20 or 24 h, then typsinised and reseeded in six-well plates, 2 × 10^3^ cells per well and grown for 7 days. Cell colonies were stained with crystal violet (0.5% w/v) containing methanol (11% v/v) for fixing, rinsed in water, air-dried and digitally scanned. Discrete colonies of > 50 cells were counted using MetaMorph software (Molecular Devices) and expressed as a percentage of vehicle treatment.

### Data analysis and statistics

Data were analysed using ANOVA with Dunnet’s multiple comparison post-test or Student’s *t*-test as indicated. Correlation between two groups was evaluated by the Pearson’s test as indicated. GI50 concentration of APR-246 was determined using the Hill equation. Coefficient of interaction (CI) between drugs (APR-246 or erastin) and TKT knockdown (TKT siRNA) or knockout (TKT sgRNA) was calculated based on a Bliss independence model^46^ as described by others^47^. A CI < 0.8 was defined as synergistic, 0.8 to 1.2 as additive and > 1.2 as antagonistic. All statistical analyses were performed with Prism 7.02 software (GraphPad) with significance level α = 0.05.

## Supplementary Information

Supplementary Information.

Supplementary Legends.

## Data Availability

All primary data can be made available upon reasonable request.
