# Supplementary material for: Transketolase regulates sensitivity to APR-246 in p53-null cells independently of oxidative stress modulation
Source: Sci Rep. 2021 Feb 24;11:4480. doi: 10.1038/s41598-021-83979-3 (PMC7904805; doi:10.1038/s41598-021-83979-3)
Supplement: Supplementary file 1 — Supplementary Information. [file 41598_2021_83979_MOESM1_ESM.pdf]

Supplementary Figures, Tables and information (full western blot images) relating to:

Transketolase regulates sensitivity to APR-246 in p53-null cells independently of oxidative stress modulation

Julia V. Milne[1,2] Bonnie Z. Zhang[1,2] Kenji M. Fujihara[1,2] Swati Dawar[1,2] Wayne A. Phillips[1,2,3] Nicholas J. Clemons[1,2]

1 Peter MacCallum Cancer Centre, 305 Grattan St, Melbourne, Victoria, Australia, 3000

2 Sir Peter MacCallum Department of Oncology, The University of Melbourne, Parkville, Victoria, Australia, 3010

3 Department of Surgery (St Vincent's Hospital), The University of Melbourne, Parkville, Victoria, Australia, 3010

Email: nicholas.clemons@petermac.org

Telephone: +61 3 8559 5273

Supplementary Figure 1

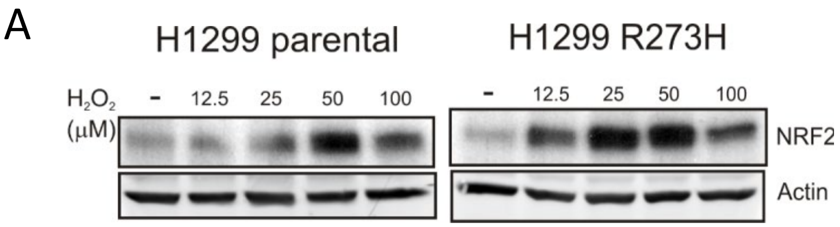

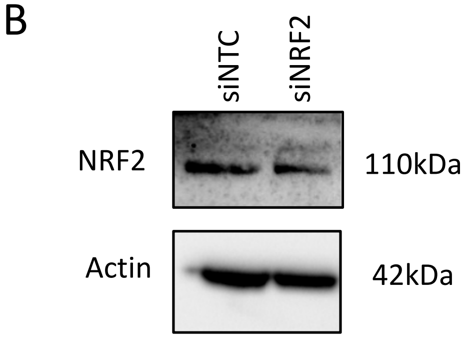

Supplementary Figure 2

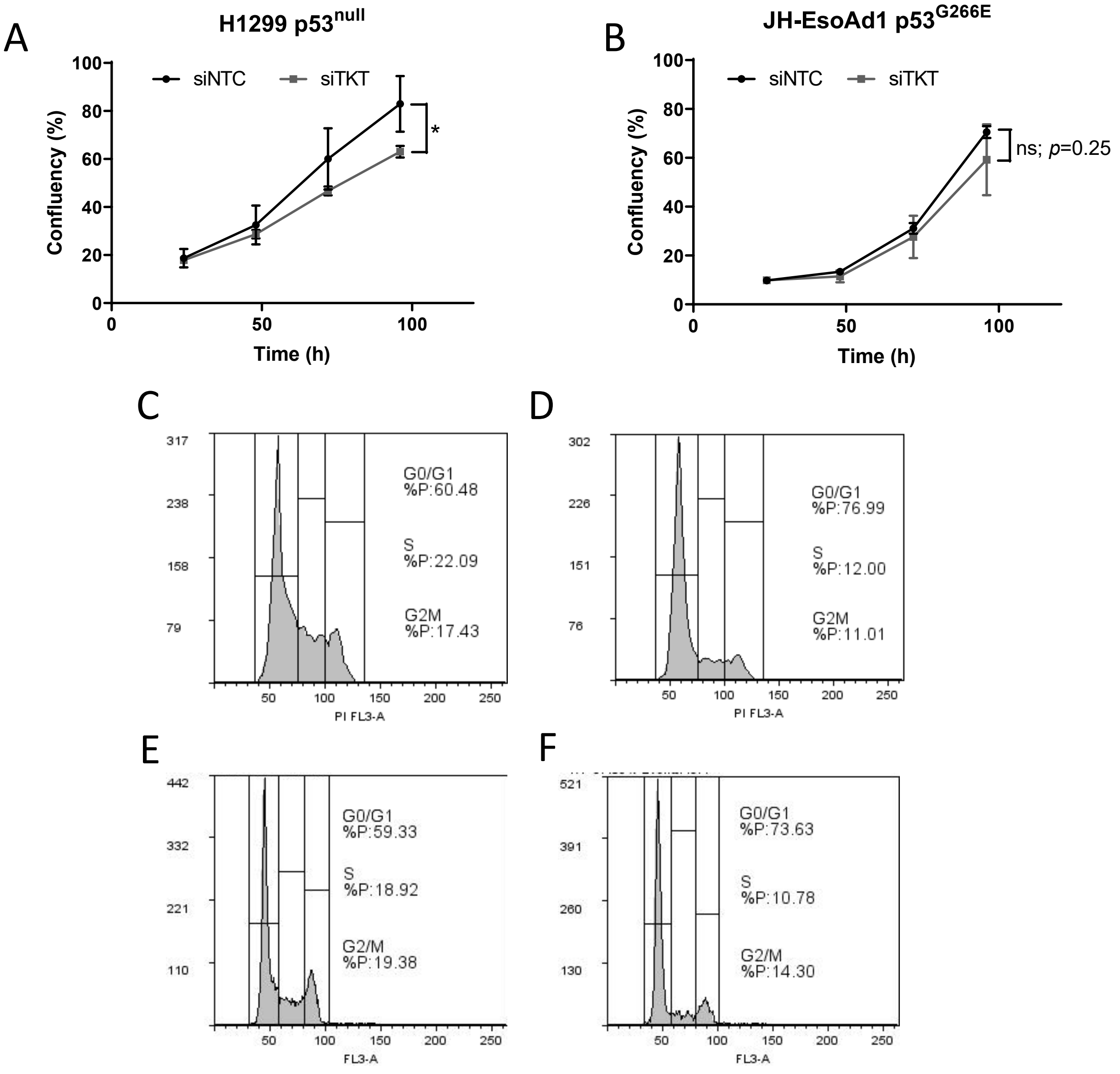

Supplementary Figure 3

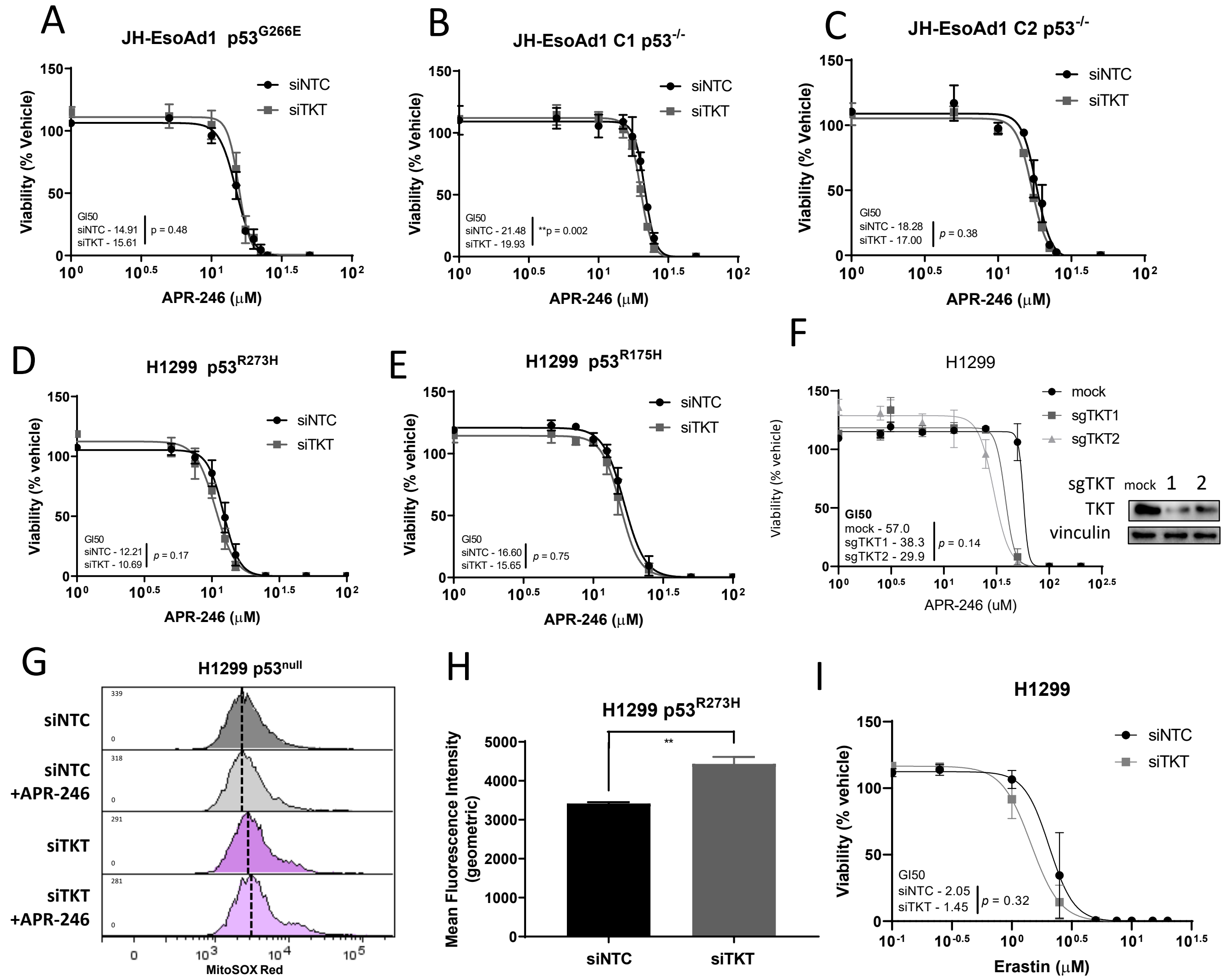

Supplementary Figure 4

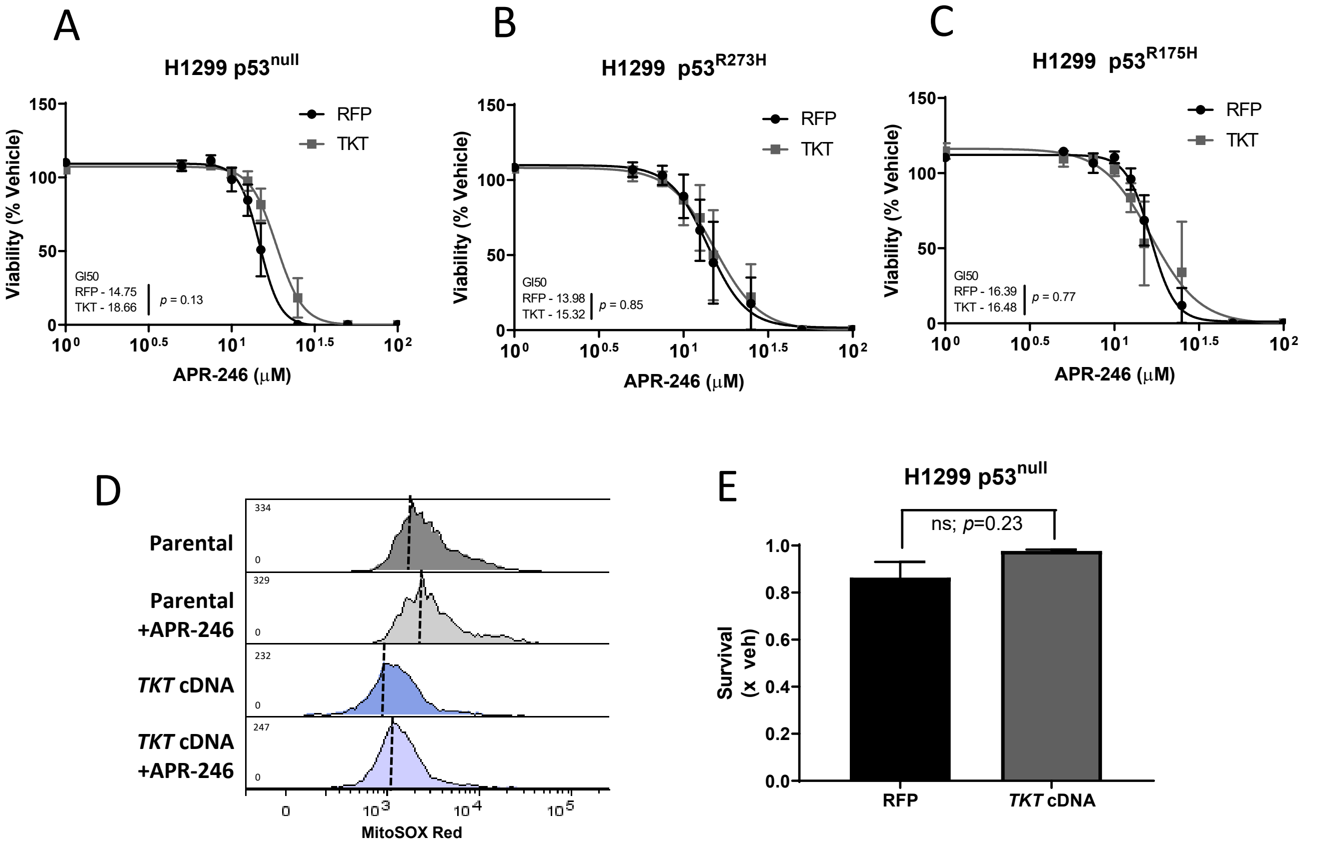

Supplementary Figure 5

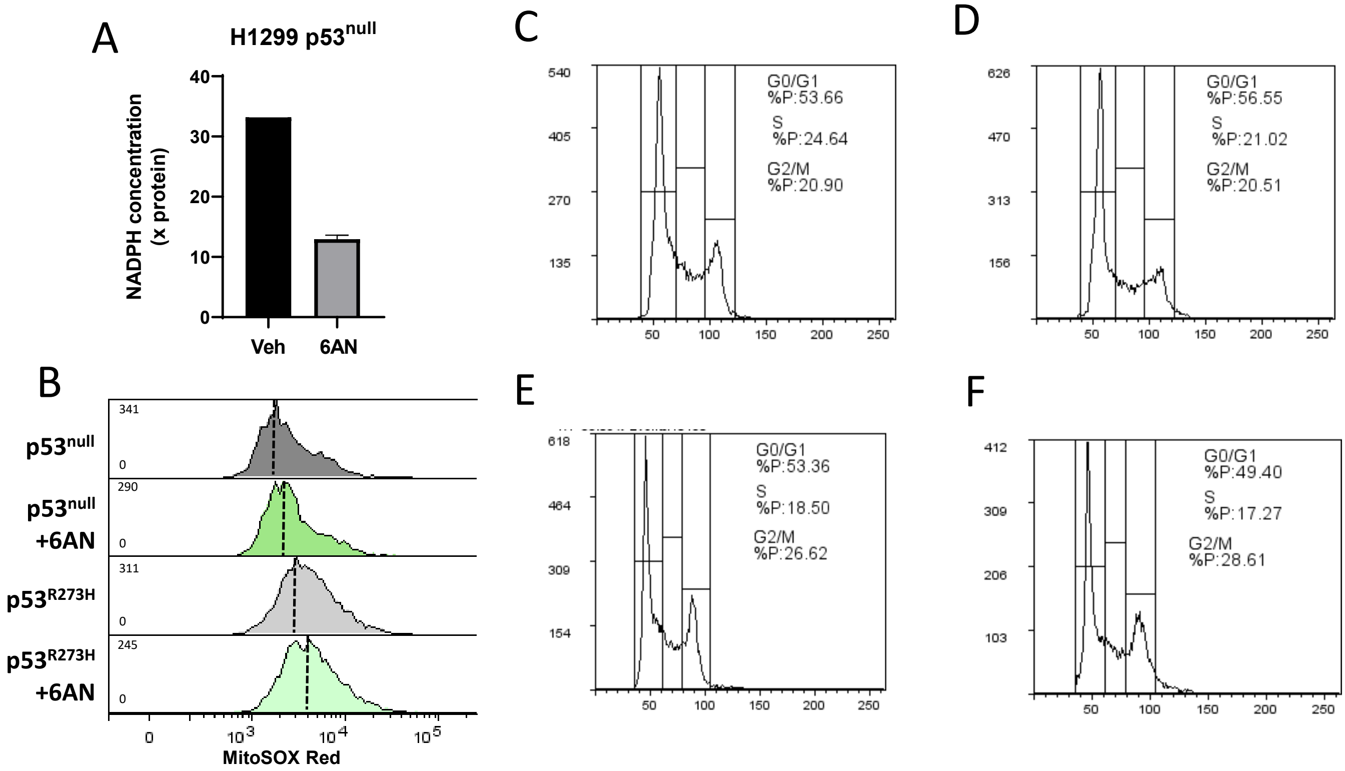

Supplementary Figure 6

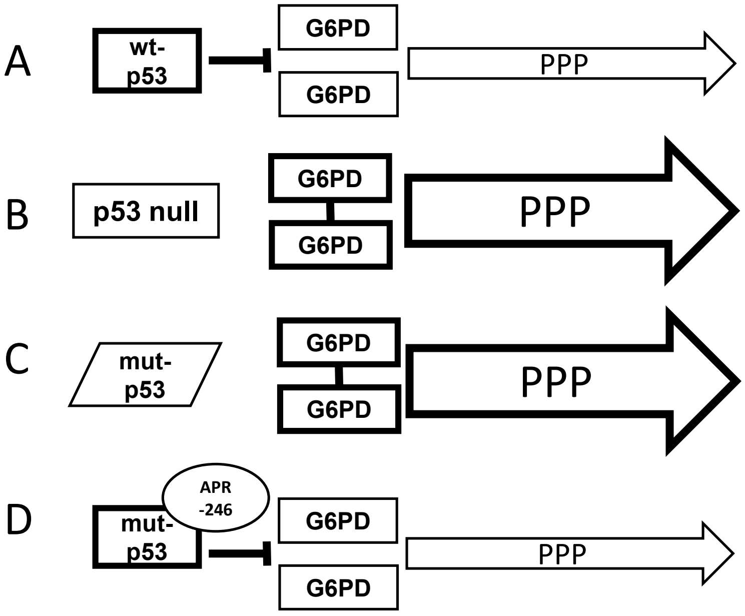

Supplementary Table 1: siRNA sequences

siRNA – (Dharmacon) ON-TARGETplus Human TKT (7086) siRNA SMARTpool (ref SO-2680077G, lot 180223)

ID	TKT (SO-2680077G)	NRF2 (M-003755-02)
1	GGAACUAGCCGCCAAUACA	GAGAAAGAAUUGCCUGUAA
2	CCGUGGAGGACCAUUAUUA	CCAAAGAGCAGUUCAAUGA
3	GCAGUUAACCGGGUACCAA	UAAAGUGGCUGCUCAGAAU
4	GAUAAGGAGUCUUGGCAUG	UGACAGAAGUUGACAAUUA

Supplementary Table 2: Real-time qPCR primer sequences

Gene	Forward (5'-3')	Reverse (5'-3')
TKT	GAAGATCAGCTCCGACTTGG	GTCGAAGTATTTGCCGGTGT
NRF2	CTTGGCCTCAGTGATTCTGAAGTG	CCTGAGATGGTGACAAGGGTTGTA
SLC7A11	ATGCAGTGGCAGTGACCTTT	GGCAACAAAGATCGGAACTG
GAPDH	GGTGTGAACCATGAGAAG	CCACAGTTTCCCGGAG

Supplementary Table 3: Antibodies for western blotting

Primary antibodies:

Antibody	Origin	Clone	Supplier	Dilution
Anti-TKT	mouse	OTI5H3	Bio-Rad	1:1000
Anti-actin	mouse	C4	MP Biomedicals	1:10000
Anti-cleaved PARP	rabbit	D214	Cell Signalling Technologies	1:1000
Anti-mutant p53	mouse	DO-1 & 1801	In-house (Haupt lab) in PBS with 3% BSA	1:20
Anti-GAPDH	mouse	6C5	Abcam	1:30000

Secondary antibodies:

Antibody	Reference	Supplier	Dilution
Polyclonal goat anti-mouse immunoglobulin/HRP	P0447	Dako	1:7000
Polyclonal swine anti-rabbit immunoglobulin/HRP	P0217	Dako	1:7000

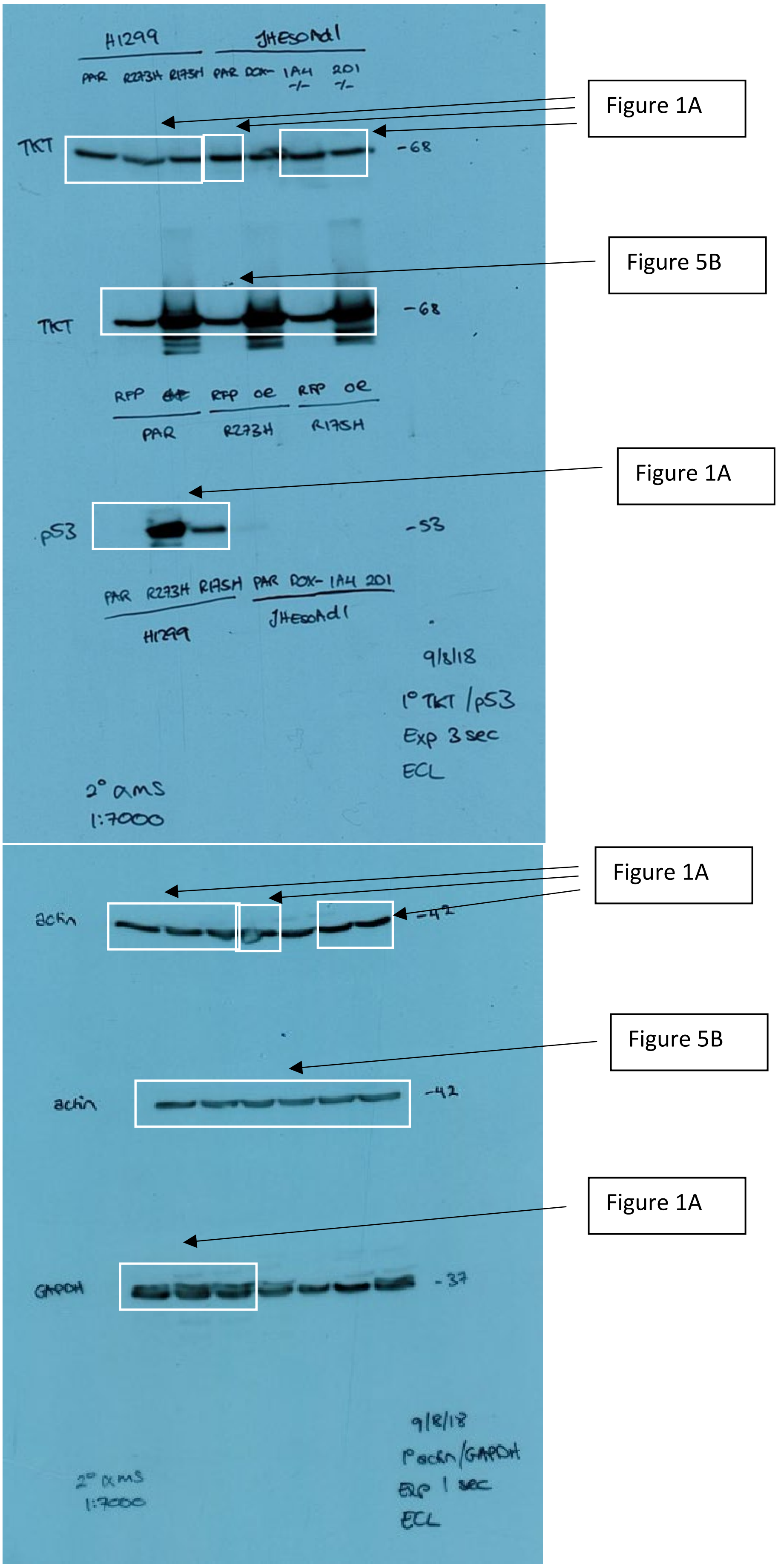
H1299
JHESOAd1
PAR R273H R175H PAR DOX- 1A4 -/- 201 -/-
Figure 1A
TKT
-68
Figure 5B
TKT
-68
RFP OE RFP OE RFP OE
PAR R273H R175H
Figure 1A
p53
-53
PAR R273H R175H PAR DOX- 1A4 201
H1299 JHESOAd1
9/8/18
1° TKT /p53
Exp 3 sec
ECL
2° αMS
1:7000
Figure 1A
actin
-42
Figure 5B
actin
-42
Figure 1A
GAPDH
-37
9/8/18
Pactin/GAPDH
Exp 1 sec
ECL
2° αMS
1:7000

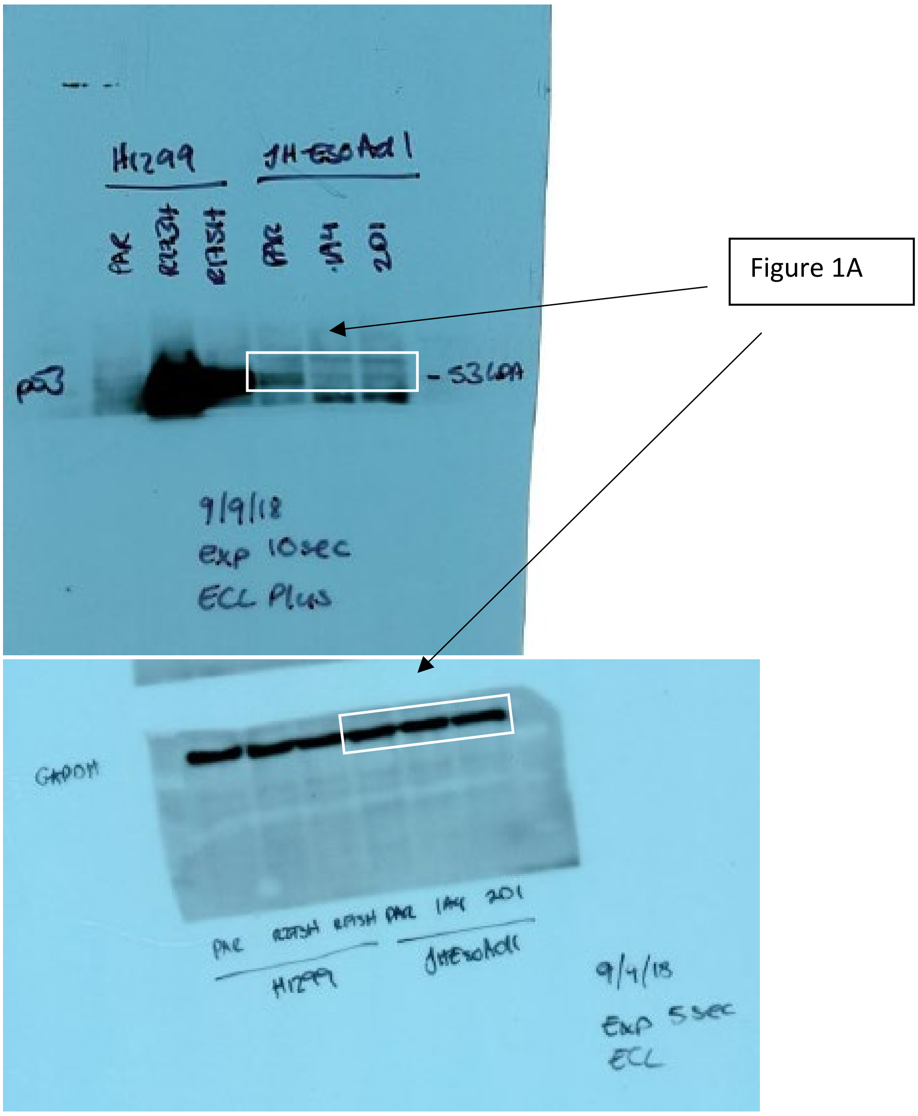
Figure 1A
H1299
JH-EsoAd1
PAR
R273H
R175H
PAR
1A4
201
p53
- 53kDa
9/9/18
Exp 10sec
ECL Plus
GAPDH
PAR
R273H
R175H
PAR
1A4
201
H1299
JH-EsoAd1
9/4/18
Exp 5sec
ECL

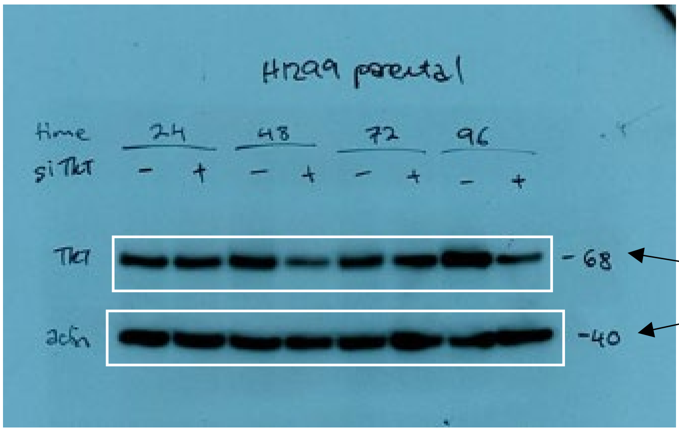

Figure 2B

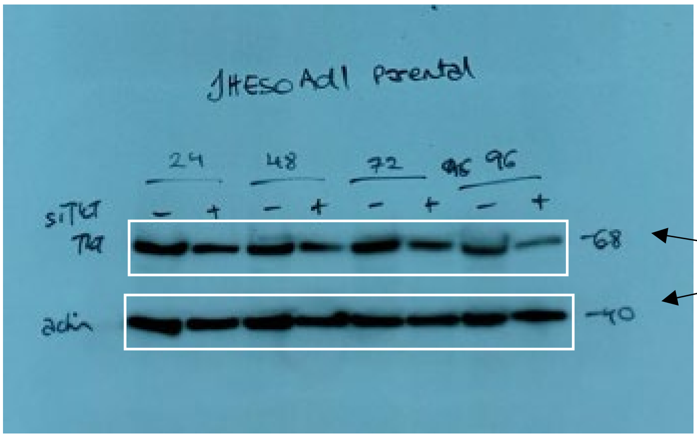

Figure 2B

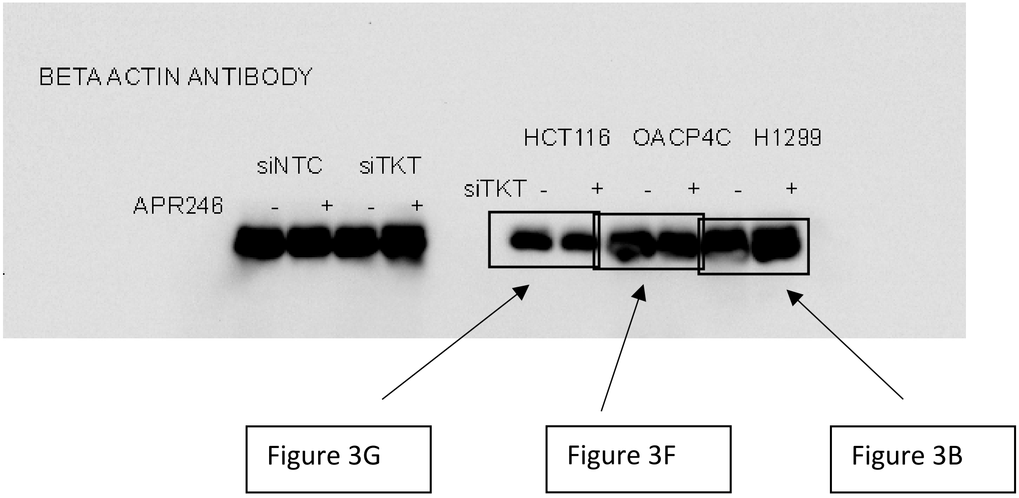
BETA ACTIN ANTIBODY
siNTC
siTKT
APR246 - + - +
HCT116
OACP4C
H1299
siTKT - + - + - +
Figure 3G
Figure 3F
Figure 3B

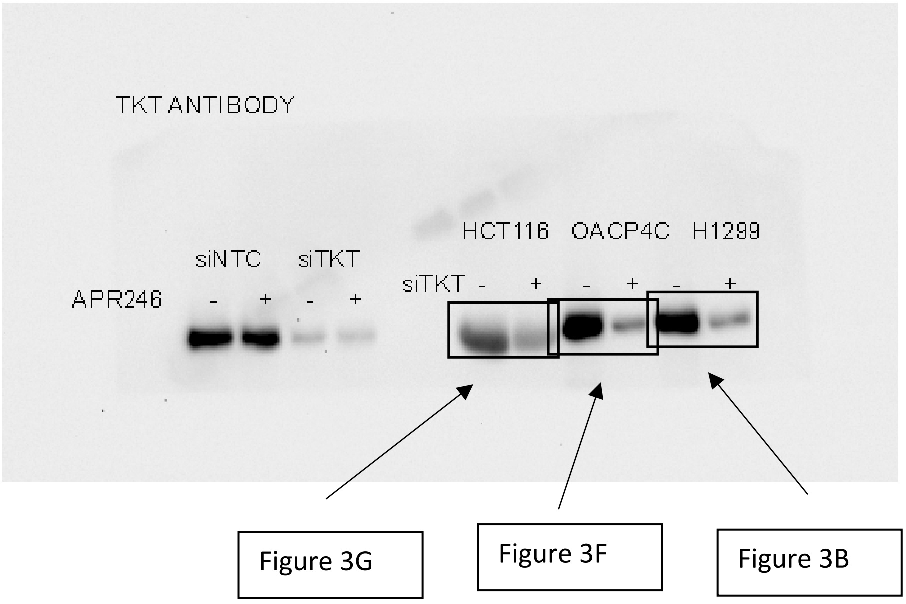
TKT ANTIBODY
siNTC
siTKT
APR246 - + - +
HCT116
OACP4C
H1299
siTKT - + - + - +
Figure 3G
Figure 3F
Figure 3B

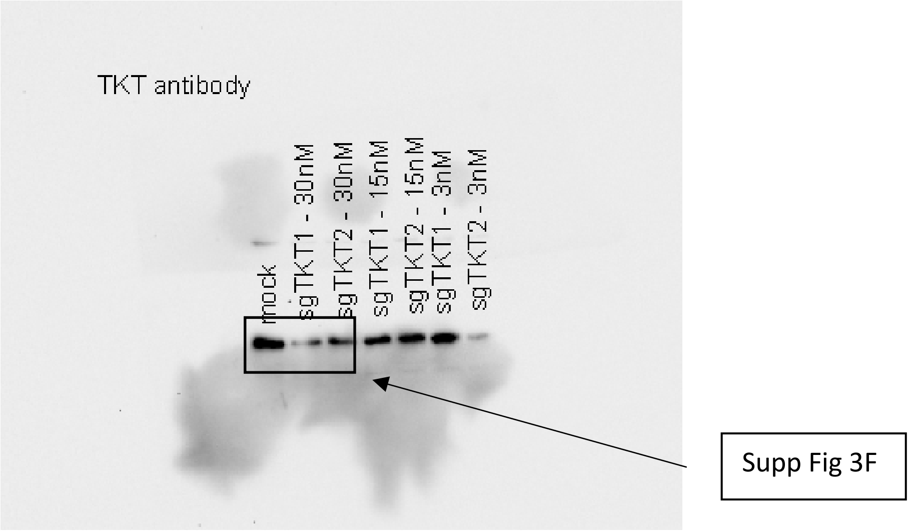
TKT antibody
mock
sgTKT1 - 30nM
sgTKT2 - 30nM
sgTKT1 - 15nM
sgTKT2 - 15nM
sgTKT1 - 3nM
sgTKT2 - 3nM
Supp Fig 3F

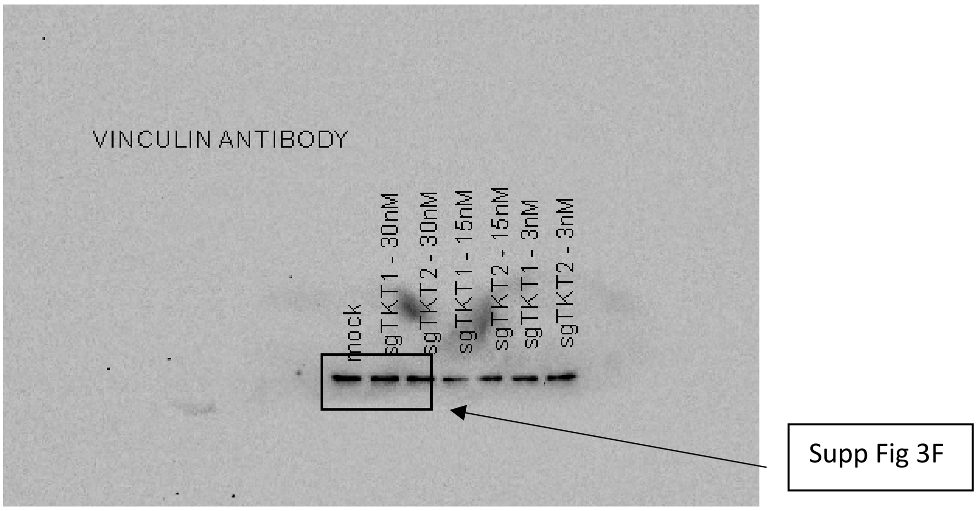
VINCULIN ANTIBODY
mock
sgTKT1 - 30nM
sgTKT2 - 30nM
sgTKT1 - 15nM
sgTKT2 - 15nM
sgTKT1 - 3nM
sgTKT2 - 3nM
Supp Fig 3F

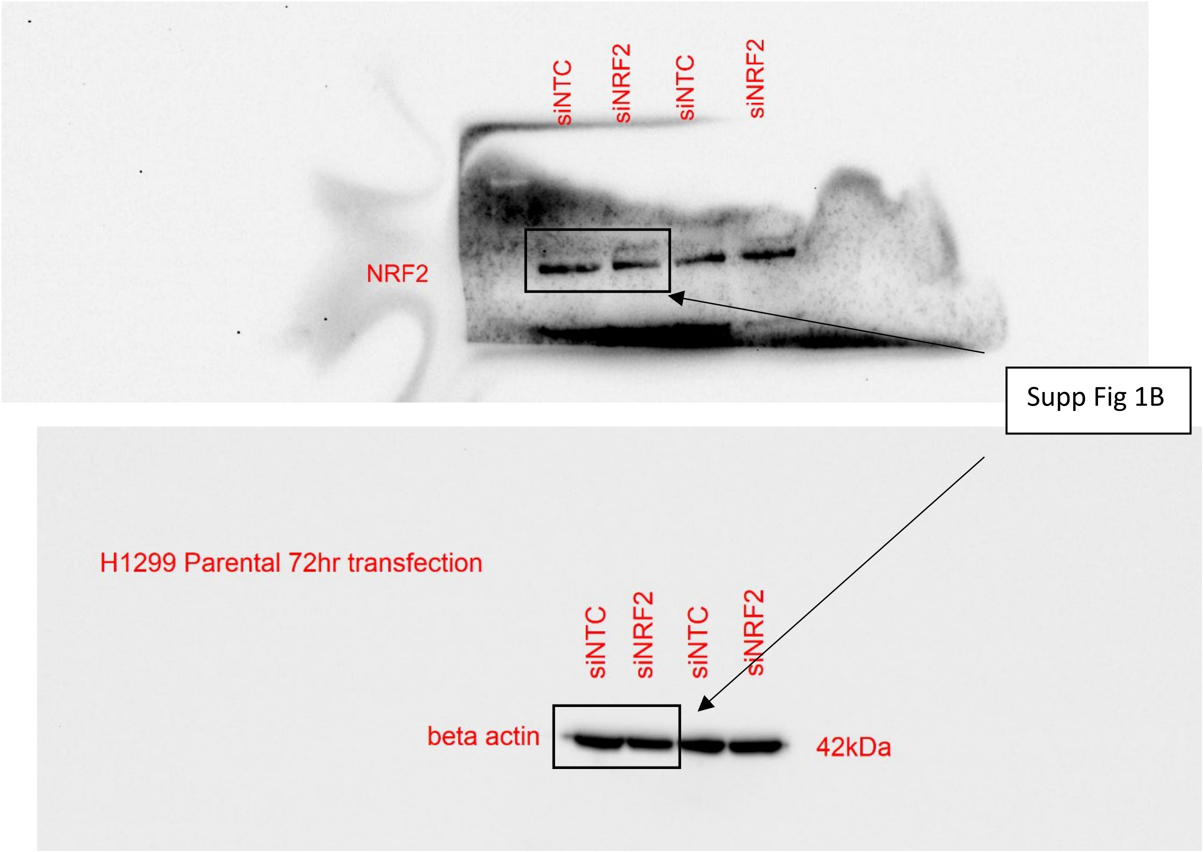
siNTC
siNRF2
siNTC
siNRF2
NRF2
Supp Fig 1B
H1299 Parental 72hr transfection
siNTC
siNRF2
siNTC
siNRF2
beta actin
42kDa
