# Supplementary material for: Transketolase regulates sensitivity to APR-246 in p53-null cells independently of oxidative stress modulation
Source: Sci Rep. 2021 Feb 24;11:4480. doi: 10.1038/s41598-021-83979-3 (PMC7904805; doi:10.1038/s41598-021-83979-3)
Supplement: Supplementary file 2 — Supplementary Legends. [file 41598_2021_83979_MOESM2_ESM.docx]

**Supplementary Figure 1:**

Expression of NRF2 protein in (A) H1299 parental (p53^null^) and p53^R273H^ cells following treatment with increasing concentrations of hydrogen peroxide (H_2_O_2_) for 4 h and (B) H1299 parental cells 72hr after transfection with siRNA targeting NRF (siRNF2) or a non-targeting control (siNTC). Blots are representative of 3 independent experiments.

**Supplementary Figure 2:**

Proliferation of (**A**) H1299 parental p53^null^ and (B) JH-EsoAd1 parental p53^G266E^ cells in vitro, representative curves as assessed by an Incucyte optical scanner following *TKT* knockdown with siRNA. Data represent mean, error bars = SD, n=3 for all studies. (**A,B**) Unpaired Student’s *t*-test. Representative flow cytometry analysis showing intensities of propidium iodide (PI) in (**C,D**) H1299 parental (p53^null^) and (**E,F**) p53^R273H^ cells following 96 h treatment with (**C,E**) non-targeting siRNA control and (**D,F**) *TKT* knockdown with siRNA.

**Supplementary Figure 3:**

Cell viability (AlamarBlue) in **(A**-C**)** JH-EsoAd1 and **(D-E)** H1299 cells following 72 h treatment with increasing concentrations of APR-246, administered 24 h after *TKT* knockdown with siRNA, normalised to vehicle control (siNTC, non-targeting control; siTKT, *TKT* siRNA **(F) (left)** Cell viability (AlamarBlue) in Cas9-expressing H1299 **c**ells following *TKT* knockout via 48 h transfection with synthetic single guide RNAs (sgRNAs) and 72 h treatment with increasing concentrations of APR-246, normalised to mock sgRNA control (mock, mock sgRNA control; sgTKT, *TKT* sgRNA **(right)** Western blots for TKT in Cas9-expressing H1299 cells with mock or *TKT*-targeting sgRNAs, showing vinculin as a loading control.**(G)** Representative flow cytometry analysis showing intensity of MitoSOX Red in H1299 parental (p53^null^) cells following 18 h APR-246 (25 µM) treatment administered after 72 h *TKT* knockdown with siRNA (siNTC, non-targeting control; siTKT, *TKT* siRNA), where Y-axis indicates cell count for each sub-panel, X-axis indicates MitoSOX fluorescence, indicative of ROS. **(H)** Geometric mean fluorescence intensity of MitoSOX Red in H1299 p53^R273H^) cells following 96 h *TKT* knockdown with siRNA, measured with flow cytometry (siNTC, non-targeting control; siTKT, *TKT* siRNA). **(I)** Cell viability (AlamarBlue) in H1299 **c**ells following 72 h treatment with increasing concentrations of Erastin, administered 24 h after *TKT* knockdown with siRNA, normalised to vehicle control (siNTC, non-targeting control; siTKT, *TKT* siRNA. Data represent mean, error bars = SEM, n=3 for all studies with the exception of **(A-C)** where n=2 . **(H)** Unpaired Student’s t-test.

**Supplementary Figure 4:**

Cell viability (AlamarBlue) in (**A**) H1299 parental (p53^null^), (**B**) p53^R273H^ and (**C**) p53^R175H^ cells with either *TKT* or red fluorescent protein (RFP) overexpression and increasing concentrations of APR-246 for 72 h. (**D**) Representative flow cytometry analysis of MitoSOX Red in H1299 parental (p53^null^) cells either untransfected (Parental) or overexpressing *TKT* (*TKT* cDNA) with 18 h APR-246 treatment (25 µM); Y-axis indicates cell count for each sub-panel, X-axis indicates MitoSOX fluorescence, indicative of ROS. (**E**) Clonogenic survival of H1299 parental p53^null^ cells with either *TKT* or red fluorescent protein (RFP) overexpression following 24 h APR-246 (50 µM) treatment. Data represent mean, error bars = SEM, n=3 for all studies excluding (**A**) where n=5 and (**E**) where n=2. (**E**) Unpaired student’s t-test. ns, not significant.

**Supplementary Figure 5:**

**(A)** Concentration of NADPH, normalised to total protein concentration, in H1299 parental (p53^null^) cells following 72 h 6AN (10 µM) treatment. (**B**) Representative flow cytometry analysis of MitoSOX Red in H1299 parental (p53^null^) and p53^R273H^ cells following 72 h treatment with 6AN (10 µM) or vehicle measured by flow cytometry. Representative flow cytometry analysis showing intensities of propidium iodide (PI) in (**C,D**) H1299 parental (p53^null^) and (**E,F**) p53^R273H^ cells following 24 h treatment with (**C,E**) vehicle or (**D,F**) 6AN. Data represent mean, error bars = SEM, n=3 for all studies. (**A**) Unpaired Student’s t-test. ns, not significant.

**Supplementary Figure 6:**

Hypothesis schematic: (**A**) Wild-type p53 (wt-p53) inhibits dimerization of glucose-6-phosphate dehydrogenase (G6PD) and reduces flux through the pentose phosphate pathway (PPP). Cells (**B**) lacking p53 (p53 null) or (**C**) with mutant p53 (mut-p53) can dimerize and thus have intact PPP. (**D**) APR-246 induces a conformational change in mut-p53, restoring a wild-type-like function, inhibiting G6PD dimerization and decreasing PPP flux.
